# LKB1 inactivation promotes epigenetic remodeling-induced lineage plasticity and antiandrogen resistance in prostate cancer

**DOI:** 10.1038/s41422-024-01025-z

**Published:** 2025-01-02

**Authors:** Fei Li, Pengfei Dai, Huili Shi, Yajuan Zhang, Juan He, Anuradha Gopalan, Dan Li, Yu Chen, Yarui Du, Guoliang Xu, Weiwei Yang, Chao Liang, Dong Gao

**Affiliations:** 1 Key Laboratory of Multi-Cell Systems, Shanghai Key Laboratory of Molecular Andrology, Center for Excellence in Molecular Cell Science, Shanghai Institute of Biochemistry and Cell Biology, Chinese Academy of Sciences, Shanghai, China; 2 University of Chinese Academy of Sciences, Beijing, China; 3 Shanghai Institute of Thoracic Oncology, Shanghai Chest Hospital, Shanghai Jiao Tong University School of Medicine, Shanghai, China; 4 Human Oncology and Pathogenesis Program, Department of Medicine, Memorial Sloan Kettering Cancer Center, New York, NY USA; 5 Key Laboratory of Systems Health Science of Zhejiang Province, School of Life Science, Hangzhou Institute for Advanced Study, University of Chinese Academy of Sciences, Hangzhou, Zhejiang China; 6 Department of Urology, The First Affiliated Hospital of Nanjing Medical University, Nanjing, Jiangsu China; 7 Institute of Cancer Research, Shenzhen Bay Laboratory, Shenzhen, Guangdong China

**Keywords:** Prostate cancer, Cancer therapeutic resistance

## Abstract

Epigenetic regulation profoundly influences the fate of cancer cells and their capacity to switch between lineages by modulating essential gene expression, thereby shaping tumor heterogeneity and therapy response. In castration-resistant prostate cancer (CRPC), the intricacies behind androgen receptor (AR)-independent lineage plasticity remain unclear, leading to a scarcity of effective clinical treatments. Utilizing single-cell RNA sequencing on both human and mouse prostate cancer samples, combined with whole-genome bisulfite sequencing and multiple genetically engineered mouse models, we investigated the molecular mechanism of AR-independent lineage plasticity and uncovered a potential therapeutic strategy. Single-cell transcriptomic profiling of human prostate cancers, both pre- and post-androgen deprivation therapy, revealed an association between liver kinase B1 (LKB1) pathway inactivation and AR independence. LKB1 inactivation led to AR-independent lineage plasticity and global DNA hypomethylation during prostate cancer progression. Importantly, the pharmacological inhibition of TET enzymes and supplementation with S-adenosyl methionine were found to effectively suppress AR-independent prostate cancer growth. These insights shed light on the mechanism driving AR-independent lineage plasticity and propose a potential therapeutic strategy by targeting DNA hypomethylation in AR-independent CRPC.

## Introduction

Epigenetic regulation plays a crucial role in determining the fate of cancer cells and their capability to undergo lineage transitions by altering the expression of genes critical for defining cellular identity and state, thus affecting the diversity within tumors and their responses to treatments.^1^ Lineage plasticity, the ability of the cells to change their identities and acquire new biological characteristics, has been recognized as a key factor in the development of resistance to targeted therapies, particularly in cancer contexts.^2–4^ In prostate cancer, the androgen receptor (AR) signaling pathway is essential for the proliferation and survival of luminal cancer cells, making androgen deprivation therapy (ADT) initially beneficial for patients. However, majority of patients will progress to a deadly form known as castration-resistant prostate cancer (CRPC). While neuroendocrine prostate cancer (NEPC) represents a well-studied example of lineage plasticity within CRPC, with its potential drivers and therapeutic approaches being increasingly identified,^5–10^ most AR-null tumors do not exhibit neuroendocrine phenotype and are instead classified as double-negative prostate cancer (DNPC),^11^ which is associated with the worst clinical outcomes among CRPC subtypes.^12^ The molecular mechanisms driving DNPC remain largely unexplored, significantly impeding the development of effective therapeutic options for DNPC.

In this study, we demonstrated that the inactivation of LKB1 pathway induced lineage plasticity and created a vulnerability to DNA methylation restoration in prostate cancer. By conducting single-cell transcriptome analysis on human prostate cancer samples before and after ADT, we investigated the molecular and cellular responses to ADT. Our findings indicated that attenuated LKB1 pathway activity was linked to AR independence in prostate cancer cells. Deletion of LKB1 promoted AR-independent lineage transformation, as well as global DNA hypomethylation in prostate cancer. Importantly, targeting DNA hypomethylation through pharmacological inhibition of TET enzymes or supplementation with endogenous metabolite S-adenosyl methionine (SAM) was shown to effectively inhibit AR-independent CRPC growth. Collectively, these results underscored the intricate relationship between metabolic dysregulation and epigenetic remodeling in driving lineage plasticity and supporting the tumorigenic expansion of AR-independent prostate cancer.

## Results

### Single-cell transcriptomics reveals the impact of ADT on lineage plasticity in human prostate cancer

**Fig. 1:**
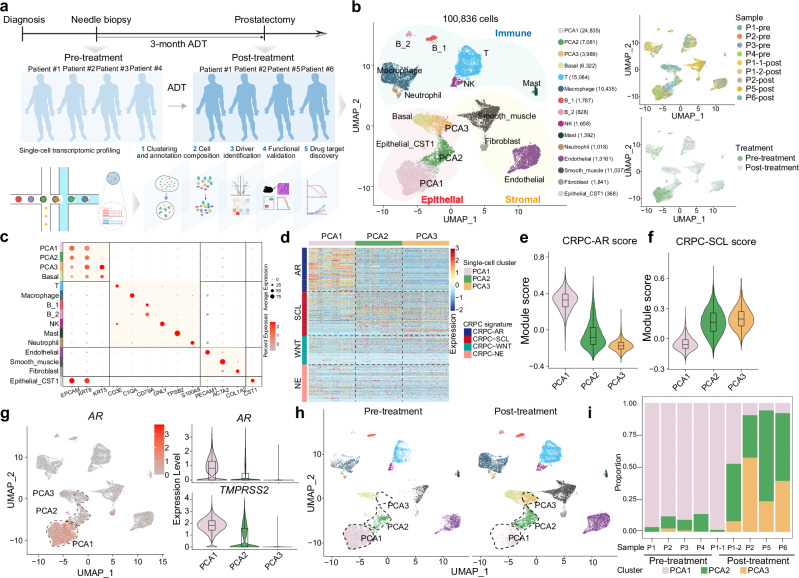
Single-cell transcriptome profiling of the ADT-induced lineage plasticity. **a** Schematic diagram of the experimental design and downstream analysis pipeline to identify the driving forces of lineage plasticity and potential therapeutic strategies in AR-independent CRPC. **b** Uniform manifold approximation and projection (UMAP) showing the information of 15 single-cell clusters comprising a total of 100,836 cells, the specimens and the treatments. **c** Dot plot showing the expression levels of the representative markers of each cell lineage. **d** Heatmap showing the module scores of the four CRPC subtypes, including CRPC-AR, CRPC-SCL, CRPC-WNT and CRPC-NE in PCA1, PCA2 and PCA3. **e**, **f** Violin plot showing the module scores of CRPC-AR (**e**) and CRPC-SCL (**f**) calculated by the *AddModuleScore* function implanted in Seurat. **g** Feature plot showing the expression level of *AR* (left) and violin plot showing the expression levels of *AR* and *TMPRSS2* (right). **h** UMAP plot according to the treatment information. **i** Cell proportions of prostate cancer cells in each of the nine specimens before or after ADT treatment.

The transition to AR-null lineage, underscored by the rising prevalence post the introduction of AR-targeting therapy like enzalutamide and abiraterone, highlights the role of AR inhibition in driving this change.^11^ To dissect the impact of ADT on prostate cancer at single-cell level, we collected fresh prostate cancer specimens from patients both before (pre-treatment, via needle biopsy) and after (post-treatment, via radical prostatectomy) 3-month ADT treatment (Fig. 1a; Supplementary information, Table S1). The pathological examination of specimens demonstrated that all of them were prostate cancer. Through single-cell RNA sequencing (scRNA-seq), we analyzed nine specimens from six patients, including paired pre- and post-treatment specimens from two individuals, capturing a comprehensive dataset of 100,836 qualified cells across epithelial, immune and stromal compartments (Fig. 1b, c; Supplementary information, Fig. S1a).

We identified three distinct clusters within prostate tumor cells: PCA1, PCA2, and PCA3 (Fig. 1b). Given the classification of CRPC into AR-dependent (CRPC-AR) and neuroendocrine (CRPC-NE) types, alongside two AR-negative/-low subtypes (CRPC-SCL and CRPC-WNT),^13^ we assessed the identity of these clusters by calculating module scores for each subtype based on signature gene expressions (Fig. 1d). The PCA1 cluster exhibited higher CRPC-AR module score compared to PCA2 and PCA3 clusters (Fig. 1e), suggesting its classification as an AR-positive prostate cancer (ARPC) subtype. Conversely, PCA2 and PCA3 demonstrated significantly higher CRPC-SCL module scores (Fig. 1f), aligning them more closely with the AR-negative/-low CRPC subtype. The module scores for CRPC-NE and CRPC-WNT were minimal across all these three clusters (Supplementary information, Fig. S1b, c). Further analysis of *AR* expression revealed high levels in PCA1, while PCA2 and PCA3 showed low to negligible expression, consistent with the expression patterns of AR target genes (*TMPRSS2*, *KLK3*, and *NKX3-1*) (Fig. 1g; Supplementary information, Fig. S1d, e). Additionally, the absence of significant *ENO2* expression across all these three cell clusters confirmed a lack of neuroendocrine differentiation (Supplementary information, Fig. S1f).

In all five post-treatment specimens, there was a significant decrease in the proportions of total prostate tumor cells among all cell types, indicating the initial clinical efficacy of ADT (Supplementary information, Fig. S1g). Notably, a closer examination of the cellular makeup within the prostate tumor cells revealed that in four out of the five post-treatment specimens, there was an increase in the cell proportions of PCA2 and PCA3 cells, along with a decrease in the proportion of PCA1 cells (Fig. 1h, i). Further trajectory inference analysis suggests that PCA1 could transform into PCA2 and PCA3 (Supplementary information, Fig. S1h–j). Given the CRPC-SCL cell identity and the lack of AR expression in PCA2 and PCA3, these findings suggest that cells within these clusters may exhibit some level of resistance to ADT. Collectively, these observations highlight a shift in the prostate tumor landscape following ADT, moving from AR-positive prostate cancer (PCA1) towards AR-negative/-low prostate cancer (PCA2 and PCA3).

### Attenuated LKB1 pathway activity associates with AR independence in human prostate cancer

**Fig. 2:**
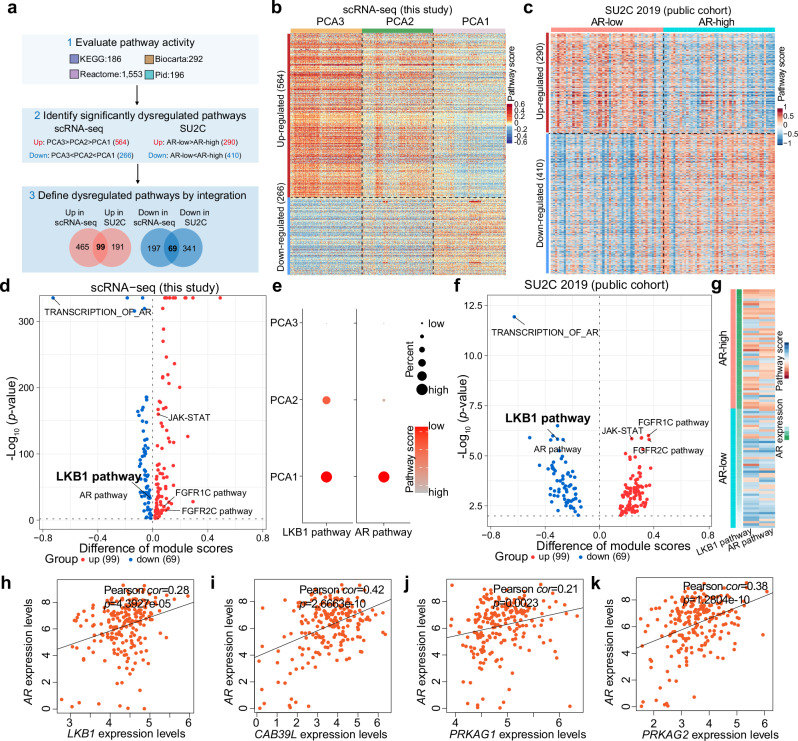
Integration analysis uncovers the attenuated LKB1 pathway activity in AR-independent human prostate cancer. **a** Scheme of the computational pipeline to systematically identify the dysregulated signaling pathways in AR-independent prostate cancer. **b** Heatmap showing the module scores of dysregulated biological pathways across PCA1, PCA2 and PCA3 cells. **c** Heatmap showing the module scores of dysregulated biological pathways across AR-low and AR-high samples. **d** Volcano plot showing the 99 upregulated and 69 downregulated pathways based on the scRNA-seq data of this study. **e** Dot plot showing the activities of LKB1 pathway (REACTOME_ENERGY_DEPENDENT_REGULATION_OF_MTOR_BY_LKB1_AMPK) and AR pathway (REACTOME_ACTIVATED_PKN1_STIMULATES_TRANSCRIPTION_OF_AR_ANDROGEN_RECEPTOR_REGULATED_GENES_KLK2_AND_KLK3) based on the scRNA-seq data of this study. **f** Volcano plot showing the 99 upregulated and 69 downregulated pathways based on a publicly available bulk RNA-seq cohort, SU2C. **g** Heatmap showing the activities of LKB1 and AR pathways based on the publicly available bulk RNA-seq cohort, SU2C. **h**–**k** Pearson correlation analysis between the expression of *AR* and the LKB1 pathway component genes, *LKB1* (**h**), *CAB39L* (**i**), *PRKAG1* (**j**) or *PRKAG2* (**k**).

To explore pathways potentially linked with the transition to AR-null lineage in prostate cancer, we developed a computational pipeline leveraging our scRNA-seq data and the publicly accessible SU2C CRPC cohort^14^ (Fig. 2a). Firstly, within the SU2C cohort, we categorized 52 samples with high *AR* expression (AR-high) (top quartile, 52/208) and 52 with low *AR* expression (AR-low) (bottom quartile, 156/208) (Supplementary information, Fig. S2a, b). As expected, AR target genes such as *NKX3-1*, *FKBP5* and *TMPRSS2* were significantly less expressed in the AR-low group (Supplementary information, Fig. S2c–e). Consistent with the basal differentiation observed in some AR-low or -negative CRPCs,^13,15^ the AR-low group showed increased expression of basal differentiation markers *TP63*, *KRT5*, and *KRT6B* (Supplementary information, Fig. S2f–h). The neuroendocrine phenotype was similarly low across these two groups (Supplementary information, Fig. S2i–k).

Secondly, we assessed the activity of 2227 signaling pathways (including KEGG: 186; Biocarta: 292; Reactome: 1553; and Pid: 196) across individual tumor cells in our scRNA-seq data and tumor samples in the SU2C cohort (Supplementary information, Table S2). This analysis identified 564 pathways upregulated (PCA3 > PCA2 > PCA1, negatively associated with AR expression) and 266 pathways downregulated (PCA3 < PCA2 < PCA1, positively associated with AR expression) from our scRNA-seq data (Fig. 2b). In the SU2C cohort, 290 pathways were upregulated in AR-low samples compared to AR-high samples (AR-low > AR-high), and 410 pathways were downregulated (AR-low < AR-high) (Fig. 2c).

Lastly, integrating these dysregulated pathways from both datasets, we identified 99 upregulated and 69 downregulated pathways showing consistent directional changes across datasets (Fig. 2d, f). Among these pathways, we observed expected dysregulations: AR-related pathways were downregulated, while JAK-STAT pathway^16^ and FGF-related pathways^11^ were upregulated (Fig. 2d, f), validating the capability of our computational pipeline to pinpoint dysregulated pathways associated with AR-null lineage plasticity. Notably, LKB1 pathway activity was consistently downregulated in both our scRNA-seq (Fig. 2d, e) and the SU2C cohort (Fig. 2f, g). Further analysis of pathway components revealed a positive correlation between the expression levels of *LKB1* (also known as *STK11*) and *AR* (Pearson correlation coefficient = 0.28; *P* = 4.3927 × 10^–5^) (Fig. 2h; Supplementary information, Fig. S2l). In addition, other LKB1 pathway components, such as *CAB39L* (Fig. 2i; Supplementary information, Fig. S2m), an activator of LKB1,^17^ and *PRKAG1* (Fig. 2j; Supplementary information, Fig. S2n) and *PRKAG2* (Fig. 2k; Supplementary information, Fig. S2o), two downstream effectors, were significantly downregulated in AR-low prostate cancers. Furthermore, the association between LKB1 pathway inactivation and AR independence was consistently validated in two additional publicly available datasets^15,18^ (Supplementary information, Fig. S2p, q). Collectively, our integrated analysis suggest that attenuated LKB1 pathway activity is linked with AR independence in human prostate cancers.

### LKB1 loss promotes prostate cancer progression

**Fig. 3:**
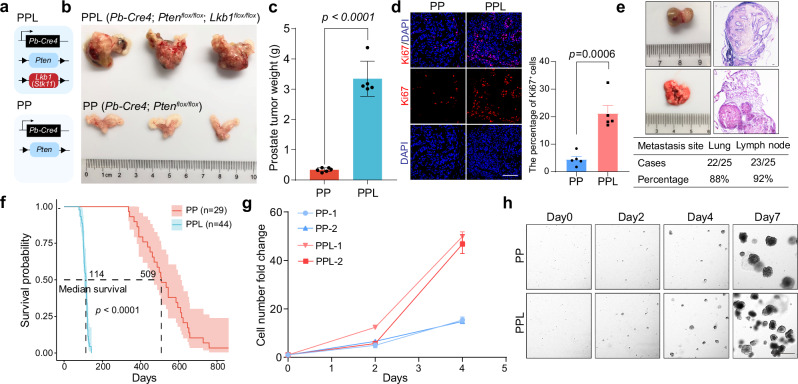
LKB1 loss promotes prostate cancer progression and metastasis. **a** Schematic diagram of construction strategy of *Pb-Cre4*; *Pten*^*flox/flox*^; *Lkb1*^*flox/flox*^ mouse model. **b** Gross anatomy of representative prostate tumors of the 15-week-old PPL and PP mice. **c** Tumor weight quantification of prostate tumors of the 15-week-old PPL and PP mice. **d** Immunofluorescence staining of DAPI and Ki67 in PP and PPL prostate tumors (left) and the quantification of the percentage of Ki67^+^ cells in total cells (right). Scale bar, 50 μm.** e** Gross anatomy of representative metastases to lung and lymph node (top) and quantification (bottom). **f** Survival statistics of PP and PPL mice. **g** Cell growth of the organoids derived from freshly dissociated tumor cells of PP and PPL mice. **h** Organoid formation of freshly dissociated tumor cells of PP and PPL mice. Scale bar, 50 μm.

To functionally validate the role of LKB1 pathway in prostate cancer progression, we engineered a genetic model by interbreeding *Lkb1*^*flox/flox*^ mice with those carrying *Pb-Cre4* and floxed *Pten* alleles (*Pb-Cre4*; *Pten*^*flox/flox*^, PP), which resulted in a prostate epithelium-specific deletion of both *Lkb1* and *Pten* (*Pb-Cre4*; *Pten*^*flox/flox*^; *Lkb1*^*flox/flox*^, PPL) (Fig. 3a). We found that the absence of LKB1 remarkably promoted prostate tumor burden (Fig. 3b, c) and enhanced tumor cell proliferation (Fig. 3d). Moreover, LKB1 loss substantially increased the propensity for prostate cancer to metastasize to the lung (88%) and lymph node (92%) in a PTEN-null context (Fig. 3e). In alignment with these findings, the PPL mice exhibited a markedly reduced survival rate in comparison to the PP mice (Fig. 3f). The capacity for organoid formation from freshly isolated PPL cancer cells was significantly higher than that of PP cancer cells (Fig. 3g, h), further confirming the tumorigenic potential induced by LKB1 loss in prostate cancer.

### LKB1 loss promotes prostate cancer lineage plasticity and antiandrogen resistance

**Fig. 4:**
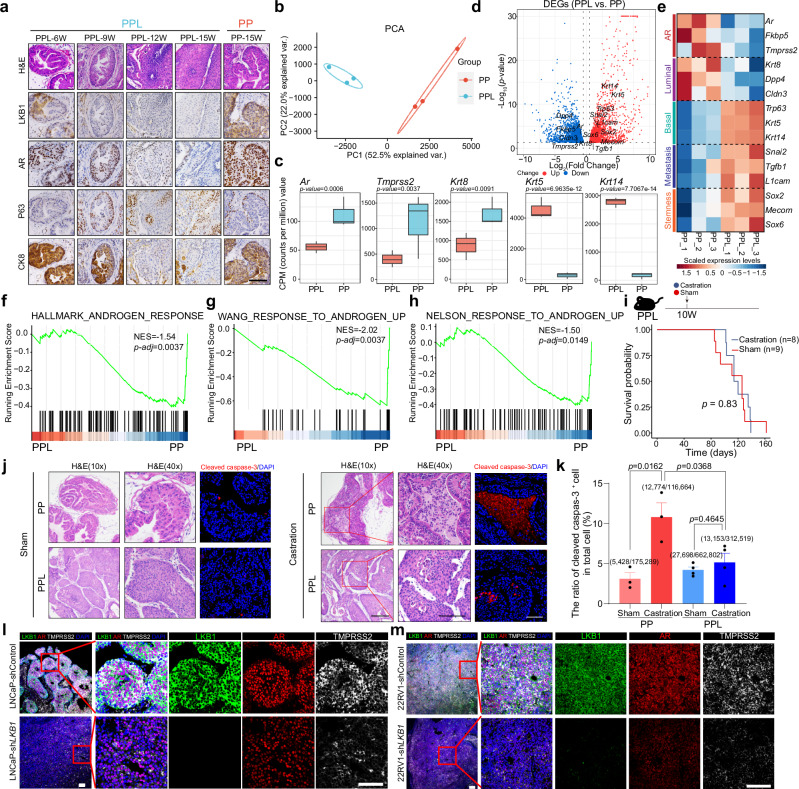
LKB1 loss confers AR-independent lineage transition in prostate cancer. **a** H&E staining and immunohistochemical staining of LKB1, AR, P63 and CK8 in the prostates of 6-, 9-, 12- and 15-week-old PPL mice and 15-week-old PP mice. **b** PCA analysis of bulk RNA-seq of the prostate tumors of 15-week-old PP and PPL mice. **c** Box plot showing the CPM (counts per million) values of *Ar*, *Tmprss2*, *Krt8*, *Krt5* and *Krt14* in the prostate tumors of 15-week-old PP and PPL mice. **d** Volcano plot showing the upregulated and downregulated DEGs between PPL and PP tumors. **e** Heatmap showing the RNA expression of three representative genes for AR, luminal, basal, metastasis and stemness phenotypes. **f**–**h** GSEA showing that compared with PP, PPL exhibited significantly lower activity of androgen-AR pathways based on three gene sets, including HALLMARK_ANDROGEN_RESPONSE (**f**), WANG_RESPONSE_TO_ANDROGEN_UP (**g**) and NELSON_RESPONSE_TO_ANDROGEN_UP (**h**). **i** Survival of PPL mice receiving sham or castration treatment starting from 10 weeks old. **j** Immunofluorescence staining of DAPI and cleaved caspase-3 in sham- or castration-treated PP and PPL prostate tumors. **k** Quantification analysis of the ratio of cleaved caspase 3^+^ cells in total cells in sham- or castration-treated PP and PPL prostate tumors. **l** Immunofluorescence staining of LKB1, AR, TMPRSS2 and DAPI in LNCaP-derived xenografts with or without *LKB1* knockdown. **m** Immunofluorescence staining of LKB1, AR, TMPRSS2 and DAPI in 22RV1-derived xenografts with or without *LKB1* knockdown. Scale bars, 50 μm.

Subsequently to evaluating the role of LKB1 inactivation, we delved into its impact on prostate cancer lineage plasticity by comparing lineage marker gene expression between PP and PPL tumors. We noted that accompanying PPL tumor progression, there was a gradual decline in the expression of AR and the luminal marker CK8, alongside an increase in the expression of the basal marker P63 (Fig. 4a). At late stages (12- and 15-week-old), PPL tumors exhibited a mixed lineage, characterized by both AR-low and AR-negative focal differentiation (Supplementary information, Fig. S3a, b). These results suggest that LKB1 inactivation triggers a lineage shift from AR-positive prostate cancer at early stages to AR-negative/-low prostate cancer at late stages.

Further analysis of differentially expressed genes (DEGs) was conducted using bulk RNA-seq on the prostate tumors from 15-week-old PPL and PP mice (Supplementary information, Table S3). Principal component analysis (PCA) revealed a distinct transcriptomic divergence between PPL and PP tumors (Fig. 4b). In line with immunohistochemistry findings, LKB1 inactivation significantly downregulated the expression of *Ar* and its target genes (*Fkbp5* and *Tmprss2*) as well as the luminal marker *Krt8* (Fig. 4c–e). Gene set enrichment analyses (GSEA) across three different gene sets uniformly indicated a significant suppression of the androgen pathway in PPL tumors (Fig. 4f–h). The upregulation of multiple lineage plasticity genes, including basal (*Trp63*, *Krt5* and *Krt14*), metastasis (*Snai2*, *Tgfb1* and *L1cam*), and stemness (*Sox2*, *Mecom* and *Sox6*) markers was induced by LKB1 loss (Fig. 4c–e), further supporting the enhanced cancer cell state plasticity by LKB1 loss. Neuroendocrine lineage genes such as *Syp*, *Foxa2*, and *Ascl1* were minimally expressed in both PP and PPL tumors (Supplementary information, Fig. S3c), suggesting a lack of neuroendocrine differentiation. Moreover, we reintroduced LKB1 expression in PPL cancer cells, and demonstrated the restored AR phenotype by LKB1 expression, underscoring the role of LKB1 in regulating lineage plasticity (Supplementary information, Fig. S3d–g).

To further corroborate the lineage plasticity induced by LKB1 inactivation, single-cell transcriptomic profiling was performed on prostate tumors from two 15-week-old PPL mice (Supplementary information, Fig. S4a). Following quality control, a total of 11,374 qualified cells spanning epithelial, stromal, and immune compartments were identified across samples (Supplementary information, Fig. S4b–d). The prostate tumor cells were categorized into three clusters: Tumor_cell_1, Tumor_cell_2 and Tumor_cell_3. An examination of lineage marker expression was conducted to ascertain the cell lineage within these tumor cell clusters. Tumor_cell_1 was found to highly express *Ar*, whereas Tumor_cell_2 and Tumor_cell_3 exhibited low or negligible *Ar* expression (Supplementary information, Fig. S4e, f). The expression of *Tmprss2* and AR signature scores displayed a corresponding pattern across these clusters (Supplementary information, Fig. S4e, f). Conversely, the basal markers, *Krt5* and *Trp63*, were predominantly expressed in Tumor_cell_2 and Tumor_cell_3 instead of Tumor_cell_1 (Supplementary information, Fig. S4f). Furthermore, analysis of trajectory inference suggested that AR-low/-negative Tumor_cell_2 and Tumor_cell_3 originated from Tumor_cell_1 (Supplementary information, Fig. S4g–i). Thus, these scRNA-seq data validated the lineage plasticity induced by LKB1 loss in prostate cancer.

The lineage transition to an AR-independent state is closely linked with the resistance to AR-targeted therapy. To investigate whether the lineage transition induced by LKB1 inactivation also correlates with a response to castration, we subjected 10-week-old PPL mice to castration and monitored their survival. The results revealed that castration did not improve the survival of PPL mice, confirming the castration resistance imparted by the loss of LKB1 (Fig. 4i). Unlike in PP tumor, where a significant increase in the percentage of cleaved caspase 3^+^ cells was observed post castration, PPL tumor showed no significant change in the level of cleaved caspase 3^+^ cells following castration (Fig. 4j, k). These results indicate that LKB1 loss endows prostate cancer with resistance to AR pathway inhibition, aligning with the lineage plasticity induced by LKB1 loss.

Furthermore, to assess the impact of LKB1 inactivation in human prostate cancer, we conducted *LKB1* knockdown experiments in two human ARPC cell lines, LNCaP and 22RV1. Consistent with findings from mouse study, knockdown of *LKB1* significantly reduced the expression of *AR* and *TMPRSS2* in xenografts derived from both LNCaP and 22RV1 cells (Fig. 4l, m). These results collectively suggest that LKB1 inactivation facilitates an AR-independent lineage transition in both human and mouse prostate cancers.

To further explore the role of LKB1 in the presence of PTEN, we generated the *Pb-Cre4*; *Lkb1*^*flox/flox*^ (PL) mouse model (Supplementary information, Fig. S5a). While LKB1 deletion alone led to prostate cancer with a low incidence, the resulting cancerous regions displayed an AR-low phenotype, characterized by the downregulation of AR (Supplementary information, Fig. S5b, c). This underscores the lineage plasticity regulated solely by LKB1 deletion. Additionally, we crossed *Lkb1*^*flox/flox*^ mice with mice carrying both *Tmprss2*^*CreERT2*^ (a luminal cell-specific inducible CreERT2 previously established by our lab^19^) and floxed Pten (*Tmprss2*^*CreERT2/CreERT2*^; *Pten*^*flox/flox*^, TP) to initiate cancer specifically in prostate luminal cells following tamoxifen administration (*Tmprss2*^*CreERT2/CreERT2*^; *Pten*^*flox/flox*^; *Lkb1*^*flox/flox*^, TPL) (Supplementary information, Fig. S5d). Despite the low knockout efficiency of LKB1 in the prostates of TPL mice, compared with the LKB1-intact regions in both TPL and TP prostates, the LKB1-deleted region in TPL prostates distinctly exhibited the AR-low phenotype (Supplementary information, Fig. S5e). These genetically engineered mouse model studies further corroborate that LKB1 inactivation is able to suppress the AR pathway in prostate cancer.

We noticed that the Thr172 phosphorylation level of AMP-activated protein kinase (AMPK) was remarkably downregulated by LKB1 loss (Supplementary information, Fig. S6a). To further clarify whether the effects of LKB1 loss on lineage plasticity is associated with AMPK or not, we introduced exogenous expression of AMPK in PPL cancer cells. We found that AMPK overexpression enhanced AR and luminal phenotype, while attenuated basal phenotype (Supplementary information, Fig. S6b–h). In addition, pharmacological activation of AMPK by the benzimidazole derivative compound 991 (C991) also showed the consistent effects on lineage plasticity (Supplementary information, Fig. S6i–o). Thus, these data demonstrated that the effects of LKB1 loss on lineage plasticity are mediated via AMPK to some degree.

### DNA hypomethylation is globally induced by LKB1 loss

**Fig. 5:**
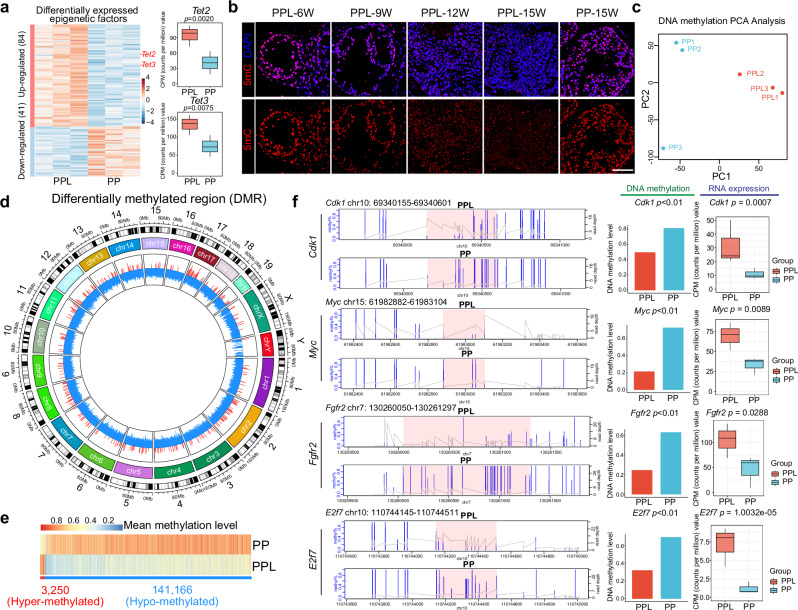
Global DNA hypomethylation is induced by LKB1 inactivation. **a** Heatmap showing the RNA expression levels of differentially expressed epigenetic factors and box plot showing the CPM values of *Tet2* and *Tet3* in PPL and PP tumors. **b** Immunofluorescence staining of 5mC and DAPI in the prostates of 6-, 9-, 12- and 15-week-old PPL mice and 15-week-old PP mice. Scale bar, 50 μm. **c** PCA analysis of WGBS data of the prostate tumors of 15-week-old PP and PPL mice. **d** Circos plot globally showing the hypo-methylated and hyper-methylated DMRs across all the chromosomes. Hypo-methylated DMRs are labeled by blue, and hyper-methylated DMRs are labeled by red. **e** Heatmap showing the methylation levels of hypo-methylated and hyper-methylated DMRs in PPL and PP tumors. **f** Methylation levels of the hypo-methylated DMRs locating at the promoter regions of *Cdk1*, *Myc*, *Fgfr2* and *E2f7* and the CPM values of these genes.

The association between lineage transition and epigenetic remodeling is well-documented across diverse biological processes.^20–22^ In the context of LKB1 loss in prostate cancer, an in-depth analysis was conducted to identify the epigenetic factors^23^ that exhibited differential expression between PPL and PP tumors. This analysis revealed 84 epigenetic factors with increased expression and 41 with decreased expression in PPL tumors compared to PP tumors (Fig. 5a; Supplementary information, Fig. S7a, b and Table S4). Among the downregulated epigenetic factors, *Foxa1*, which associates with the regulation of prostate cancer lineage plasticity, was identified (Supplementary information, Fig. S7b, c). Notably, among the upregulated epigenetic factors, *Tet2* and *Tet3* were identified (Fig. 5a), both of which play crucial roles in DNA methylation metabolism, suggesting a potential dysregulation of DNA methylation in PPL tumors.

DNA methylation, a key epigenetic modification in mammals, typically occurs at the fifth carbon of cytosine bases, resulting in 5-methylcytosine (5mC).^24^ To assess changes in DNA methylation, 5mC levels were examined through immunofluorescence staining, revealing a gradual decrease in DNA methylation correlating with cancer progression in both in vivo tumors (Fig. 5b) and in vitro organoids (Supplementary information, Fig. S7d). For a more detailed analysis of DNA methylation changes, whole-genome bisulfite sequencing (WGBS) was performed on PP and PPL tumors. PCA and clustering analyses highlighted distinct DNA methylation patterns between PP and PPL tumors (Fig. 5c; Supplementary information, Fig. S7e). Subsequent identification of differentially methylated regions (DMRs) between PPL and PP tumors (PPL vs PP) revealed 3250 hyper-methylated and 141,166 hypo-methylated DMRs (Supplementary information, Table S5), indicating a global trend of DNA hypomethylation in PPL tumors (Fig. 5d, e). Given the role of promoter DNA methylation in transcriptional regulation, DMRs within promoter regions (±3 kb around transcription start site) were specifically analyzed and integrated with RNA expression profiles. This analysis showed significant hypo-methylation in the promoter regions of *Cdk1*, *Myc*, *Fgfr2*, and *E2f7* along with higher RNA expression levels in PPL tumors compared to PP tumors (Fig. 5f). The oncogenic roles of these genes have been established in multiple cancer types^25–28^ suggesting that targeting DNA hypomethylation could be a viable therapeutic strategy in prostate cancer.

Additionally, analyses of differentially methylated loci (DMLs) at single-nucleotide resolution further confirmed the global DNA hypomethylation induced by LKB1 loss (DMLs were listed in Supplementary information, Table S6), with 4914 hyper-methylated and 360,382 hypo-methylated DMLs identified (Supplementary information, Fig. S7f, g). This reinforces the notion that LKB1 inactivation globally promotes DNA hypomethylation, potentially contributing to the observed lineage plasticity and therapeutic resistance in prostate cancer.

To explore the mechanism by which LKB1 loss induces DNA hypomethylation, we determined the levels of SAM and methionine, two key metabolites in DNA methylation metabolic pathway, in both PPL and PP tumors. We found that LKB1 loss significantly downregulated SAM levels, while methionine levels remained unaffected (Supplementary information, Fig. S8a, b), suggesting that the synthesis of SAM from methionine was affected by LKB1 loss. To elucidate how LKB1 regulates SAM levels, we conducted mass spectrometry-based analysis of the LKB1 interactome in PP tumor cells overexpressing Flag-LKB1, and finally identified a total of 176 enriched proteins (Supplementary information, Fig. S8c, d and Table S7). Notably, we discovered and further validated that LKB1 could interact with methionine adenosyltransferase 2A (MAT2A) (Supplementary information, Fig. S8d–g), a rate-limiting enzyme for the synthesis of SAM.^29^ Moreover, we introduced LKB1 expression in PPL cancer cells, and found that LKB1 expression significantly enhanced MAT2A activity (Supplementary information, Fig. S8h). These data suggest that LKB1 loss attenuates the enzymatic activity of MAT2A, leading to decreased SAM levels and consequent DNA hypomethylation.

### Sensitivity of AR-independent prostate cancer to targeting DNA hypomethylation

**Fig. 6:**
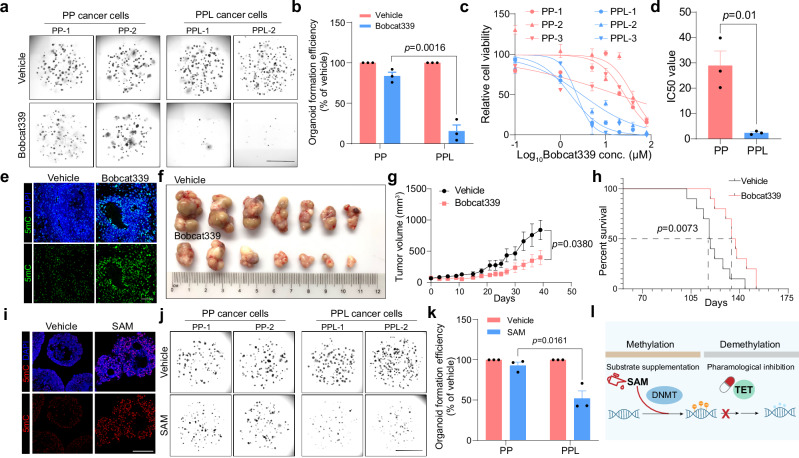
Restoring DNA methylation suppresses AR-independent prostate cancer. **a** Organoid formation of freshly dissociated tumor cells from PPL and PP mice under vehicle or Bobcat339 (15 μM) treatment condition. Scale bar, 50 μm. **b** Quantification of the organoid formation efficiency of PPL and PP cancer cells under vehicle or Bobcat339 treatment condition. **c**, **d** Drug sensitivity assay of Bobcat339 in PPL cancer organoids (**c**) and quantification (**d**). **e** Immunofluorescence staining of 5mC and DAPI in the allograft tumors derived from PPL cancer organoids under vehicle or Bobcat339 treatment condition. Scale bar, 50 μm. **f** Allografts derived from PPL cancer organoids under vehicle or Bobcat339 treatment condition. **g** Tumor growth of the allografts derived from PPL cancer organoids under vehicle or Bobcat339 treatment condition. **h** Survival of PPL mice under vehicle or Bobcat339 treatment condition. **i** Immunofluorescence staining of 5mC and DAPI in the allograft tumors derived from PPL cancer organoids under vehicle or SAM treatment condition. Scale bar, 50 μm. **j** Organoid formation of freshly dissociated tumor cells from PPL and PP mice under vehicle or SAM (50 μM) treatment condition. Scale bar, 50 μm. **k** Quantification of the organoid formation efficiency of PPL and PP cancer cells under vehicle or SAM treatment condition. **l** Schematic diagram showing targeting DNA hypomethylation by exogenous supplementation with SAM and pharmacological inhibition of TETs with Bobcat339.

Having identified the global DNA hypomethylation in AR-independent prostate cancer, we proceeded to investigate the therapeutic potential of counteracting this epigenetic alteration. In mammals, DNA demethylation predominantly occurs through TET-mediated oxidation of 5mC suggesting that inhibiting TET enzymes could promote DNA methylation. We treated PP and PPL organoids in vitro with Bobcat339, a cytosine-based inhibitor of TET.^30^ Contrary to the negligible impact on PP cancer cells, Bobcat339 remarkably suppressed the proliferation of PPL cancer cells (Fig. 6a, b). This differential sensitivity was underscored by the substantially reduced IC_50_ (half-maximal inhibitory concentration) value for Bobcat339 in PPL cancer cells (PP: 28.9 μM; PPL: 2.4 μM), indicating a pronounced vulnerability of PPL cancer cells to DNA methylation restoration (Fig. 6c, d). In vivo application of Bobcat339 not only augmented 5mC levels of PPL allograft as expected (Fig. 6e), but also markedly diminished PPL tumor growth (Fig. 6f, g) and significantly extended the survival of PPL mice (Fig. 6h).

Furthermore, we explored the effects of augmenting cellular DNA methylation through external supplementation with SAM, a primary methyl donor. SAM administration significantly increased DNA methylation levels (Fig. 6i), and unlike the minimal influence on PP cancer cells, notably inhibited the proliferation of PPL cancer cells (Fig. 6j, k). These results align with the growth-inhibitory effects of TET inhibition by Bobcat339 on PPL cells, rather than PP cells. Given the heightened sensitivity of PPL cells to Bobcat339, these findings reinforce the concept.

Collectively, these data demonstrate the dependency of PPL cancer cell growth on DNA hypomethylation, advocating for the therapeutic strategy of targeting this epigenetic vulnerability through both pharmacological inhibition of TETs and supplementation of SAM to combat AR-independent prostate cancer (Fig. 6l).

### Targeting DNA hypomethylation in aggressive prostate cancer

**Fig. 7:**
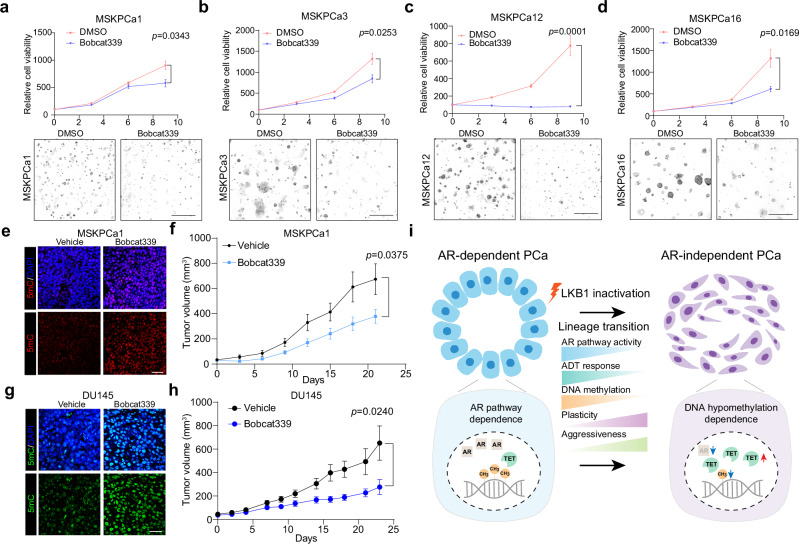
Targeting DNA hypomethylation suppresses human AR-independent prostate cancer. **a**–**d** Relative cell viability of AR-negative/-low prostate cancer organoid lines, including MSKPCa1 (**a**), MSKPCa3 (**b**), MSKPCa12 (**c**) and MSKPCa16 (**d**), under vehicle or Bobcat339 (50 μM) treatment condition. Scale bars, 50 μm. **e** Immunofluorescence staining of 5mC and DAPI in the xenografts derived from MSKPCa1 organoids under vehicle or Bobcat339 treatment condition. Scale bar, 50 μm. **f** Tumor growth of the xenografts derived from MSKPCa1 organoids under vehicle or Bobcat339 treatment condition. **g** Immunofluorescence staining of 5mC and DAPI in the xenografts derived from DU145 cells under vehicle or Bobcat339 treatment condition. Scale bar, 50 μm. **h** Tumor growth of the xenografts derived from DU145 cells under vehicle or Bobcat339 treatment condition. **i** Schematic diagram elucidating that LKB1 inactivation promotes AR-independent lineage plasticity, antiandrogen resistance and DNA hypomethylation in prostate cancer, and targeting DNA hypomethylation facilitates the development of therapeutic strategies.

Following the identification of DNA hypomethylation’s pivotal role in mouse models of prostate cancer, we aimed to extend these observations to human prostate cancer. We assessed DNA methylation status in xenografts derived from multiple human prostate cancer cell lines and organoids using 5mC immunofluorescence staining. Consistent with the findings in AR-independent mouse prostate cancers, AR-negative human prostate cancers exhibited lower levels of DNA methylation compared to AR-positive counterparts (Supplementary information, Fig. S9a). Further investigation into the therapeutic potential of Bobcat339 on a selection of AR-negative/-low prostate cancer organoid lines revealed a significant inhibition of organoid growth under in vitro condition (Fig. 7a–d). Compared to AR-positive cancer cells, AR-negative/-low prostate cancer cells were more sensitive to Bobcat339 treatment (Supplementary information, Fig. S9b, c). Similarly, in vivo studies demonstrated Bobcat339’s capacity to inhibit the growth of MSKPCa1 (Fig. 7e, f) and DU145, an LKB1-negative/AR-negative prostate cancer cell line (Fig. 7g, h).

The clinical significance of TET enzymes, including TET1, TET2, and TET3, was assessed by examining their genetic alterations in cBioPortal.^31^ We noted a higher frequency of TET alterations in metastatic castration-resistant prostate cancer (mCRPC) compared to primary prostate cancer (Supplementary information, Fig. S9d, e). Notably, the predominant type of genetic alteration differed between primary prostate cancer, where deep deletion prevailed, and mCRPC, which was characterized by a predominance of amplifications (Supplementary information, Fig. S9e). Moreover, the TET alterations in mCRPC were positively associated with the exposure to AR inhibitors, such as abiraterone and enzalutamide (Supplementary information, Fig. S9f). The association of TET upregulation with DNA hypomethylation, coupled with clinical amplification of TETs in aggressive prostate cancer, underscores the potential of targeting DNA hypomethylation as a therapeutic strategy for aggressive prostate cancer.

## Discussion

Lineage plasticity plays a crucial role in the resistance to targeted therapies across multiple cancers,^22,32^ with neuroendocrine transition in CRPC being one of the most extensively studied form of such lineage plasticity. Despite the identification of potential drivers and therapeutic interventions for this transition, it is important to acknowledge that majority of AR-null tumors do not exhibit neuroendocrine characteristics and remains poorly understood. Therefore, unraveling the molecular underpinnings and developing appropriate therapeutic strategies for AR-independent prostate cancer are urgently needed.

The frequency of lineage transition towards an AR-independent phenotype has notably risen following the introduction of the AR pathway antagonists, such as enzalutamide and abiraterone,^11,32^ hinting at a direct link between AR-null lineage development and AR pathway inhibition. To define the potential mechanisms of AR-independent prostate cancer, we conducted single-cell transcriptomic profiling of human prostate cancers pre- and post-ADT. Through these analyses, we identified dysregulated pathways in AR-negative/-low prostate cancer compared to their AR-positive counterparts. Beyond the previously reported activation of the FGFR^11^ and JAK-STAT pathways,^16^ our study also revealed an association between inactivation of LKB1 pathway and AR independence. We developed a mouse model with prostate-specific *Lkb1* knockout in a *Pten*-null background, performed histological examination and scRNA-seq analyses, and found that LKB1 loss not only promoted prostate cancer aggressiveness but also facilitated AR-independent lineage transition, consistent with a previous study.^33^ This lineage transition, driven by LKB1 loss, was characterized by a significant decrease in AR and its target genes, alongside castration resistance. Consistent with previous studies,^11,13,15^ we observed that AR-independent prostate cancer was enriched with basal, metastasis and stemness signatures.

DNA methylation plays a pivotal role in cell fate decisions during mammalian development,^24^ and alterations in DNA methylation present promising targets for developing diagnostic, prognostic, and therapeutic strategies in various cancers.^34^ In prostate cancer, aberrant DNA methylation has been identified as a key driver of lineage plasticity and resistance to AR-targeted therapy,^35–38^ with NEPC differentiation being functionally linked to DNA hypermethylation.^37^ Notably, our study observed genome-wide DNA hypomethylation in AR-independent prostate cancers, and demonstrated that reversing DNA hypomethylation through pharmacological inhibition and metabolite supplementation effectively suppressed these cancers. These findings suggest a lineage-specific dysregulation of DNA methylation in CRPC, providing a basis for lineage-specific clinical interventions by targeting DNA methylation.

Interestingly, deletion of LKB1 in the context of KRAS^G12D^ in adult pancreatic ducts leads to global DNA hypermethylation,^39^ contrasting with the association between LKB1 inactivation and DNA hypomethylation observed in our study. This discrepancy underscores the cancer-dependent or driver-dependent roles of LKB1 in DNA methylation regulation, highlighting the need for further research to elucidate the cellular and molecular basis of this difference. Such insights would be invaluable in developing targeted therapeutic strategies focusing on DNA methylation in cancers.

In conclusion, this study demonstrates that LKB1 inactivation contributes to AR-independent lineage plasticity, DNA hypomethylation and antiandrogen resistance in prostate cancer (Fig. 7i). These findings underscore the potential of targeting DNA hypomethylation as a therapeutic strategy for treating patients with CRPC.

## Materials and methods

### Mice

All mouse studies were approved by the Center for Excellence in Molecular Cell Science (CEMCS) Animal Care and Use Committee. Mice were bred and maintained according to Shanghai Laboratory Animal Center Institutional Animal Regulations. The *Lkb1*^*flox/flox*^ mice were gifted from Dr. Hongbin Ji. *Lkb1*^*flox/flox*^ mice were crossed with the mice carrying *Pb-Cre4* and floxed Pten (*Pb-Cre4*; *Pten*^*flox/flox*^, PP) alleles to obtain *Pb-Cre4*; *Pten*^*flox/flox*^; *Lkb1*^*flox/flox*^ mice (PPL). *Tmprss2*^*CreERT2/CreERT2*^; *Pten*^*flox/flox*^; *Lkb1*^*flox/flox*^ *mice* (TPL) were obtained by crossing *Lkb1*^*flox/flox*^ mice with the mice carrying both *Tmprss2*^*CreERT2*^ and floxed Pten (*Tmprss2*^*CreERT2/CreERT2*^; *Pten*^*flox/flox*^, TP). The SCID mice were purchased from LINGCHANG Biotech.

### In vitro assay

PP and PPL mouse prostate tumors were freshly dissociated into single cells. Cells were counted and in total 500 cells were seeded in a 50 μL of Matrigel drop and cultured in CO_2_ incubator (5% CO_2_, 37 °C). On the zero day, the second day, the fourth day and sixth day, organoid viability was detected with 3D cell viability assay (Promega, G9683) and organoid picture was captured. For the drug sensitivity experiment of PP and PPL organoids to Bobcat339 (TOPSCIENCE, T5198), 500 tumor cells of PP or PPL were seeded in a 50 μL of Matrigel drop in the middle of one well of 24-well suspension plate. 1 mL of mouse prostate culture medium was gently pipetted with Bobcat339 at 0 μM, 0.1 μM, 1 μM, 5 μM, 10 μM, 20 μM, 40 μM and 80 μM into each well and then the plate was placed into CO_2_ incubator (5% CO_2_, 37 °C). On the sixth day, organoid viability was detected with 3D cell viability assay and organoid picture was captured. For the drug sensitivity experiment of AR-positive prostate cancer cell lines and AR-negative/-low prostate cancer cell lines to Bobcat339, 2000 cells were seeded in a well of 96-well plate. 200 μL culture medium was gently pipetted with Bobcat339 at 0 μM, 0.1 μM1, 1 μM, 5 μM, 10 μM, 20 μM, 40 μM and 80 μM into each well and then the plate was placed into CO_2_ incubator. On the sixth day, cell viability was detected. For pharmacological activation of AMPK, we treated PPL cancer cells with an AMPK activator, the benzimidazole derivative C991 (TOPSCIENCE, TQ0028) at 10 μM for 72 h.

### In vivo assay

PPL tumor cells, MSKPCa1 or DU145 cells were transplanted to SCID mice by subcutaneous injection. Bobcat339 was freshly dissolved in the vehicle containing 5% DMSO, 40% PEG300, 5% Tween 80 and 50% H_2_O. When the tumor volume reached 100–200 mm^3^, the mice was randomly assigned to Bobcat339 at 10 mg/kg, or vehicle every other day by intraperitoneal injection.

### Determinations of SAM and methionine

Samples were prepared following the modified protocol.^40^ The frozen tissue was retrieved from either liquid nitrogen or a –80 °C freezer and allowed to thaw on ice for 1 h. Approximately 30 mg of tissue was transferred to a 1.5 mL tube. 300 μL of ice-cold methanol/water (50%/50%) containing 10 mM acetic acid was added to the tube and the tissue was finely minced using scissors. The tissue was homogenized using a tissue grinder, followed by the addition of 300 μL of chloroform. The tissue homogenate was thoroughly mixed using a rotary mixer at 4 °C for 60 min. The tissue homogenate was centrifuged at 13,000 rpm, 4 °C for 30 min, resulting in the formation of two layers: an upper aqueous phase and a lower organic phase. 200 μL of the aqueous phase was retained. After 1 h in a vacuum concentrator, the precipitate was resuspended in 50 μL of water/acetonitrile/formic acid (40%/60%/0.1%). Then, 75 μL of acetonitrile was added and the mixture was vortexed for 30 min at 4 °C. Following centrifugation at 14,000 rpm, 4 °C for 50 min, the supernatant was carefully aspirated and immediately frozen in liquid nitrogen before storage at –80 °C.

Metabolites were quantified using a 6495B Triple quadrupole mass spectrometer equipped with an ESI probe and 1260 HPLC system (Agilent, USA). BEH Amide column (100 mm × 2.1 mm id, 1.7 µm particle size, Waters) coupled with a VanGuard Pre-Column (5 mm × 2.1 mm id, 1.7 µm particle size, Waters) was employed at 25 °C and autosampler was set to 4 °C. Briefly, a binary gradient of solvent A of 5 mM ammonium formate in 90% acetonitrile containing 0.1% formic acid and solvent B of 5 mM ammonium formate in 10% acetonitrile containing 0.1% formic acid was used for gradient elution: 0–8 min, 90% A; 8–10 min, 90% A to 70% A; 10–10.1 min, 70% A to 50% A; 10.1–13 min, 50% A hold for 3 min; 13.1–20 min, 50% A to 90% A. A flow rate of 0.3 mL/min was used. The total run time was 20 min. The MS was operated in positive mode. The following parameters were optimized for metabolite analysis: drying gas temperature 250 °C, drying gas flow 11 L/min, sheath gas temperature 280 °C, sheath gas flow 11 L/min, nebulizer pressure 25 psi, capillary voltage 3500 V, fragmentor voltage 380 V. RF voltage amplitudes of high-pressure and low-pressure ion funnels are 150 V and 60 V, respectively.

### Immunoprecipitation and immunoblotting analysis

Cells were washed with PBS and lysed using a modified buffer. Whole cell lysates were then incubated overnight at 4 °C with specific antibodies. The lysates were subsequently incubated with protein A/G Sepharose beads for 2 h at 4 °C, followed by three washes with cell lysis buffer. The proteins were eluted from the beads by incubating in 2× loading buffer at 95 °C for 10 min. Eluates, along with cell lysate extracts (input samples), were separated by SDS-PAGE, transferred onto PVDF membranes, and subjected to immunoblotting.

### MAT2A enzyme activity assay

MAT2A activity was determined by measuring ATP consumption using Kinase-Glo Plus kit (Promega). MAT2A was immunoprecipitated from the cell lysates and subjected to MAT2A enzymatic assays in the reaction buffer containing 50 mM HEPES (pH 7.5), 25 mM MgCl_2_, 25 mM KCl, 500 μM L-methionine, 10 μM ATP, 1 mM DTT, and then mixing with equal volume of Kinase-Glo buffer. The luminescence of ATP was measured using EnVision Multilabel Plate Readers (Perkin Elmer, USA). The whole reaction was carried out at room temperature.

### Library construction for bulk RNA-seq

RNA was extracted from dissected prostate tumors. RNA sequencing libraries were further constructed with the VAHTS mRNA-Seq V3 Library Prep Kit for Illumina (Vazyme, NR611). The constructed libraries were sequenced by Berry Genomics on an Illumina NovaSeq 6000 (PE150) platform with up to 6 GB of data per library.

### Data processing and analyses for bulk RNA-seq

Clean reads were mapped to the mm10 genome using HISAT2 (version 2.1.0).^41^ Gene expression was further quantified at the gene level using featureCounts.^42^ Genes with *P* values less than 0.05 and absolute log_2_-fold changes greater than 0.5 (*P* < 0.05 and |Log_2_FC| >  0.5) were defined as DEGs by DESeq2.^43^ Based on androgen pathway signature genesets, GSEA analyses were performed by the *GSEA* function and the results were visualized by the *gseaplot2* function in clusterProfiler R package.^44^

### Library construction for WGBS

Genomic DNA was extracted from 20 mg of tumor tissues using QIAamp DNA Mini Kit (QIAGEN, 51306). Bisulfite conversion of 1000 ng of genomic DNA was performed using EpiArt DNA Methylation Bisulfite Kit (Vazyme, EM101-01). WGBS libraries were further prepared using EpiArt DNA Methylation Library Kit (Vazyme, NE102). The constructed libraries were sequenced by Berry Genomics on an Illumina NovaSeq 6000 (PE150) platform with up to 60 GB of data per library.

### Data processing and analyses for WGBS

Bisulfite mapping to the mm10 genome assembly and methylation calling were performed using bismark^45^ using the default parameters. DMRs were defined by the *callDMR* function (p.threshold = 0.01) and DMLs were defined by the *callDML* function (p.threshold = 0.001) implanted in DSS R package.^46^ Genomic annotations for DMRs and DMLs were performed by using the *annotatePeak* function implanted in ChIPseeker R package.^47^ To identify the potential association between the dysregulated DNA methylation and gene expression, the genes having hypomethylated DMRs in their promoter region (WGBS data) and showing upregulated expression levels (RNA-seq data) were specifically extracted.

### Library construction for scRNA-seq of human prostate cancer specimens

The single cell suspension prepared was loaded into a microfluidic device, and the scRNA-seq library was constructed according to the Singleron GEXSCOPE protocol of the GEXSCOPE Single Cell RNA Library Kit (Singleron Biotechnologies), including cell lysis, mRNA capture, cell (barcode) and mRNA (UMI) labeling, reverse transcription of mRNA to cDNA and amplification, and cDNA fragmentation. Pools were sequenced on Illumina HiSeq X at 150 bp paired-end reads.

### Data processing and analyses of scRNA-seq of human prostate cancer specimens

Raw reads from scRNA-seq were processed to generate gene expression matrices using CeleScope (version 1.9.0) (https://github.com/singleron-RD/CeleScope) pipeline. Briefly, raw reads were first processed with CeleScope to remove low-quality reads with Cutadapt (version 1.17) to trim poly-A tail and adapter sequences. Cell barcode and UMI were extracted. After that, we used STAR^48^ (version 2.6.1a) to map reads to the reference genome GRCh38. UMI counts and gene counts of each cell were acquired with featureCounts^42^ (version 2.0.1) software, which resulted in the expression matrix files for subsequent analysis.

The resulting expression matrices were further processed by using the *CreateSeuratObject* function of Seurat^49^ (version 4.0.3). Data integration and normalization, dimensionality reduction, cell clustering and annotation were also performed in Seurat (version 4.0.3). (1) The *merge* function was used to merge multiple datasets into single Seurat object. Qualified cells were characterized with nFeature_RNA > 500 & nFeature_RNA < 6000 & percent.mt < 25. (2) The *FindIntegrationAnchors* function was applied to identify anchors, followed by the *IntegrateData* function to integrate multiple datasets. (3) After data scaling with the *ScaleData* function, dimensional reduction analysis was performed by using the *RunPCA* and *RunUMAP* functions. Subsequently, the *FindNeighbors* and *FindClusters* functions were used to group cells into distinct clusters.

Increasing evidences suggest that basal differentiation can emerge in CRPC. To distinguish normal basal cells from the cancer cells with basal phenotype, CopyKAT,^50^ a computational tool specialized for the inference of genomic copy number in single-cell data, was applied to evaluate genomic copy number. Cells with genome-wide copy number aberrations are considered as prostate cancer cells, while basal cells have diploid copy number profiles.

### Library construction and data analyses of scRNA-seq of mouse prostate tumors

Prostate tumors from two 15-week-old PPL mice were dissected and minced with scissors, and further dissociated into single cell suspension as previously described. In brief, the minced tissues were enzymatically dissociated in DMEM/F12 buffer with 1× collagenase/hyaluronidase (STEMCELL, 07912) by a 30-min incubation at 37 °C. Tissues were mechanically pipetted every 10 min during incubation. The dissociated tissues were further centrifuged at 300× *g* for 2 min, and the supernatant was then removed. TrypLE (Gibco, 12605-028) supplemented with 10 μM Y27632 (Selleck, S1049) was added to suspend tissue chunks, followed by 15-min incubation at 37 °C. The digestion reaction was quenched by adding DMEM supplemented with 10% FBS, and the cell suspension was applied to a 70-μm cell strainer placed in a 15 mL tube. Centrifugation at 1700 rpm for 3 min was performed to collect cells.

The DNBelab C Series Single-Cell Library Prep Set (MGI, 940-000047-00, 16 RXN) was used to construct the scRNA-seq library of mouse prostate tumors. In brief, droplet generation, emulsion breakage, reverse transcription and cDNA amplification to generate barcoded libraries and indexed libraries were performed according to the manufacturer’s protocol. Libraries were sequenced on a MGISEQ-2000 sequencer with the following sequencing strategy: 41-bp read length for read 1 and 100-bp read length for read 2.

Raw reads were processed to generate gene expression matrices using DNBC4tools (https://github.com/MGI-tech-bioinformatics/DNBelab_C_Series_HT_scRNA-analysis-software) pipeline. Based on the raw gene expression matrices, qualified cells were characterized with nFeature_RNA > 500 & nFeature_RNA < 6000 & percent.mt < 15. The filtered expression matrices were further respectively processed by the functions of *NormalizeData*, *FindVariableFeatures*, *ScaleData*, *RunPCA*, *RunUMAP*, *FindNeighbors* and *FindClusters* implanted in the Seurat. AR signature scores in each tumor cluster were evaluated by the *AddModuleScore* function in Seurat. Trajectory inference of prostate tumor cells was performed by the functions of *slingshot* and *slingPseudotime* implanted in the Slingshot.^51^

### Identification of dysregulated pathways in AR-negative prostate cancers

To identify the potential driving forces of AR-independent lineage transition, based on scRNA-seq data in this study in combination with a publicly available CRPC cohort, SU2C, a computational pipeline was developed. (1) Based on AR expression levels in the SU2C cohort, 52 AR-high (first quarter, 52/208) and 52 AR-low (last quarter, 156/208) samples were identified, respectively. (2) A total of 2227 signaling pathways, including 186 KEGG pathways, 292 Biocarta pathways, 1553 Reactome pathways and 196 Pid pathways were collected from https://www.gsea-msigdb.org/gsea/msigdb/collections.jsp. (3) Pathway module scores of 2227 signaling pathways were evaluated in each tumor cluster of our scRNA-seq data by the *AddModuleScore* function in Seurat, and in each tumor samples of SU2C cohort by GSVA R package.^52^ (4) In our scRNA-seq data, 564 upregulated pathways (PCA3 > PCA2 > PCA1, negatively associated with AR expression) and 266 downregulated pathways (PCA3 < PCA2 < PCA1, positively associated with AR expression) were identified. In the SU2C cohort, 290 upregulated pathways (AR-low > AR-high) and 410 downregulated pathways (AR-low < AR-high) were identified. (5) Through integrating these dysregulated pathways from two datasets, 99 upregulated and 69 downregulated pathways showing consistent change direction between two datasets were finally identified.

### Clinical relevance of TET alteration

To explore the clinical relevance of TETs in human prostate cancer, the genetic alteration of TETs was analyzed by using publicly available cohorts in the cBioPortal (https://www.cbioportal.org/).^31^ The two following datasets were selected for downstream analyses: primary prostate cancer (TCGA, Firehorse Legacy) and mCRPC (SU2C/PCF Dream Team^14^). *TET1*, *TET2* and *TET3* were input as query genes. The “Cancer Types Summary” analysis was performed to compare the differences in the genetic alteration frequency and heterogeneity of these three TETs between primary prostate cancer and mCRPC. The “Comparison/Survival” analysis was performed to explore the association between the alteration status of TETs and the exposure status of abiraterone and enzalutamide in mCRPC.

### Quantification and statistical analyses

Two-tailed Student’s *t*-tests were performed with GraphPad Prism 7 and R to compare differences between two groups. The numbers of independent experiments and samples are indicated in the figure legends. Data with replicates are presented as the means ± standard errors of the means (SEMs).

Other materials and methods are described in Supplementary information, Data S1.

## Supplementary information

Supplementary information, Fig. S1

Supplementary information, Fig. S2

Supplementary information, Fig. S3

Supplementary information, Fig. S4

Supplementary information, Fig. S5

Supplementary information, Fig. S6

Supplementary information, Fig. S7

Supplementary information, Fig. S8

Supplementary information, Fig. S9

Supplementary information, Table S1

Supplementary information, Table S2

Supplementary information, Table S3

Supplementary information, Table S4

Supplementary information, Table S5

Supplementary information, Table S6

Supplementary information, Table S7

Supplementary information, Data S1

## Data Availability

The raw data for scRNA-seq, bulk RNA-seq and WGBS of mouse prostate cancer samples have been deposited in GSA (https://ngdc.cncb.ac.cn/gsa/) under CRA014136. The raw data for scRNA-seq of human prostate cancer specimens have been deposited in GSA-human (https://ngdc.cncb.ac.cn/gsa-human/) under HRA006365. All analyses in this manuscript were performed using publicly available software and code. Details and key parameter settings of each analysis have been described in the Materials and methods section.
