# Author Correction: LKB1 inactivation promotes epigenetic remodeling-induced lineage plasticity and antiandrogen resistance in prostate cancer

**DOI:** 10.1038/s41422-025-01097-5

**Published:** 2025-03-18

**Authors:** Fei Li, Pengfei Dai, Huili Shi, Yajuan Zhang, Juan He, Anuradha Gopalan, Dan Li, Yu Chen, Yarui Du, Guoliang Xu, Weiwei Yang, Chao Liang, Dong Gao

**Affiliations:** 1 Key Laboratory of Multi-Cell Systems, Shanghai Key Laboratory of Molecular Andrology, Center for Excellence in Molecular Cell Science, Shanghai Institute of Biochemistry and Cell Biology, Chinese Academy of Sciences, Shanghai, China; 2 University of Chinese Academy of Sciences, Beijing, China; 3 Shanghai Institute of Thoracic Oncology, Shanghai Chest Hospital, Shanghai Jiao Tong University School of Medicine, Shanghai, China; 4 Human Oncology and Pathogenesis Program, Department of Medicine, Memorial Sloan Kettering Cancer Center, New York, NY USA; 5 Key Laboratory of Systems Health Science of Zhejiang Province, School of Life Science, Hangzhou Institute for Advanced Study, University of Chinese Academy of Sciences, Hangzhou, Zhejiang China; 6 Department of Urology, The First Affiliated Hospital of Nanjing Medical University, Nanjing, Jiangsu China; 7 Institute of Cancer Research, Shenzhen Bay Laboratory, Shenzhen, Guangdong China

**Keywords:** Prostate cancer, Cancer therapeutic resistance

Correction to: *Cell Research* 10.1038/s41422-024-01025-z, published online 02 January 2025

It has come to our attention that in the version of the article initially published, in Fig. 6b, the dots of the bar plot representing vehicle-treated samples were not included; in Fig. 6j, the image for vehicle-treated PP-1 cells was mistakenly reused from the image for SAM-treated PP-2 cells. This was due to an inadvertent incorrect pasting or editing during figure panel organization. We have meticulously reviewed our raw data and provided the correct images for Fig. 6b and j. This correction does not affect the quantification of the results or the conclusions of this study. We sincerely apologize for this oversight.

**Figure:**
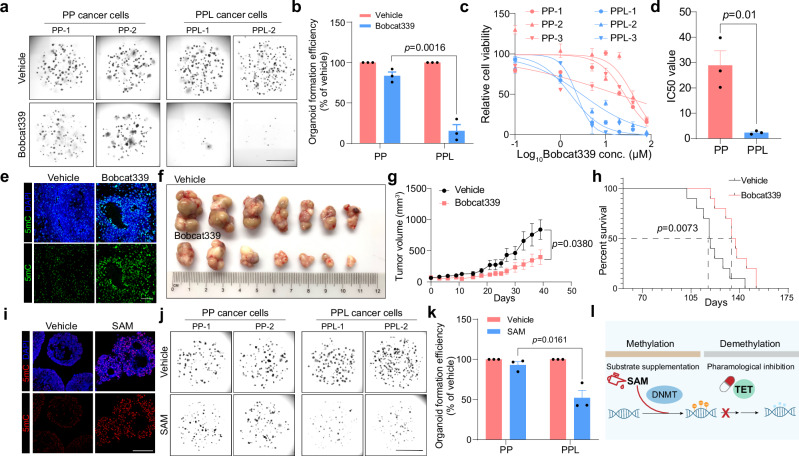

The original article has been corrected.

