# Supplementary material for: LKB1 inactivation promotes epigenetic remodeling-induced lineage plasticity and antiandrogen resistance in prostate cancer
Source: Cell Res. 2025 Jan 2;35(1):59–71. doi: 10.1038/s41422-024-01025-z (PMC11701123; doi:10.1038/s41422-024-01025-z)
Supplement: Supplementary file 1 — Supplementary information, Fig. S1 [file 41422_2024_1025_MOESM1_ESM.pdf]

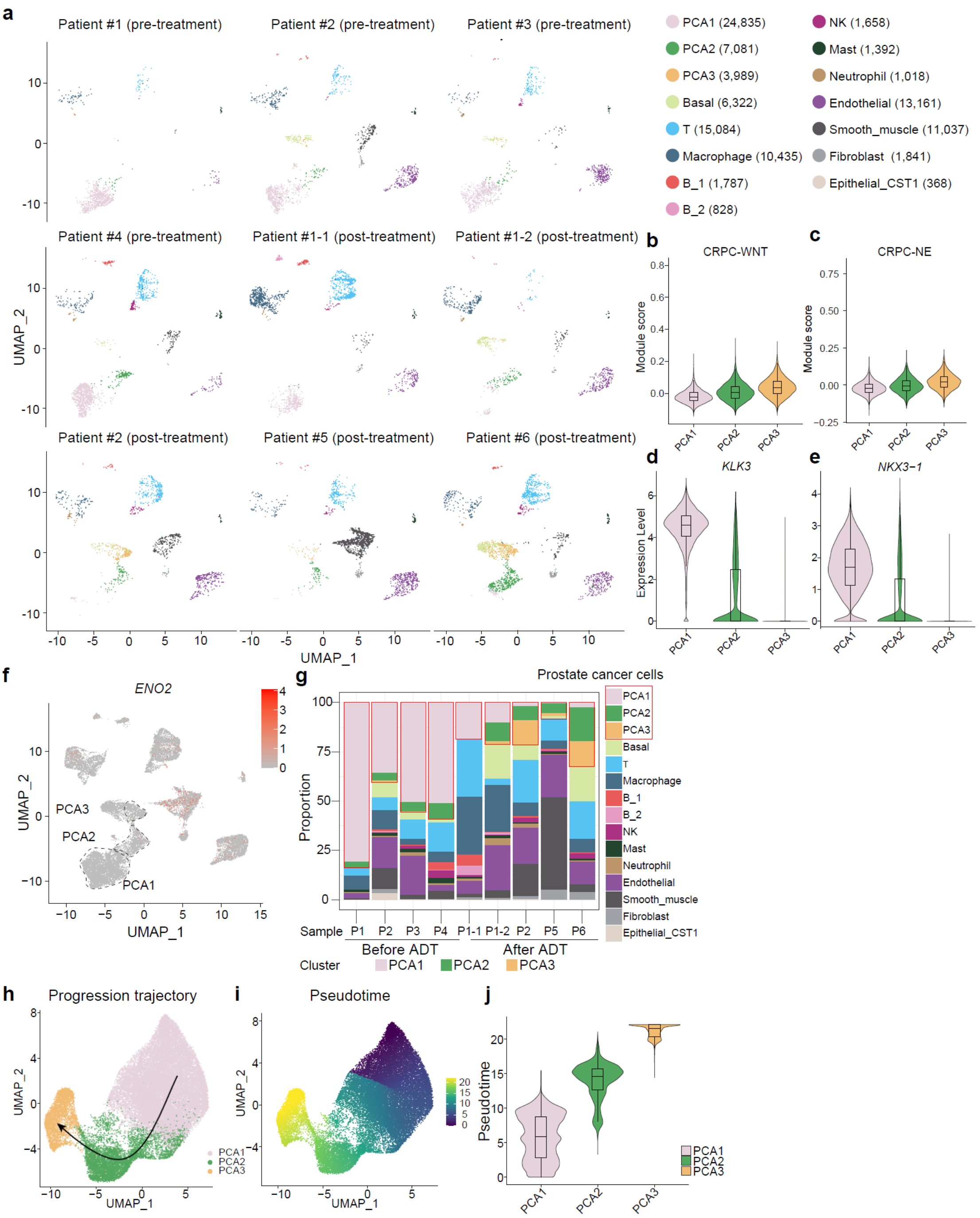

Supplementary information, Fig. S1. Single-cell RNA-seq of human prostate cancers before and after ADT treatment. a UMAP showing the single-cell clusters across the specimens. **b** Violin plot showing the module scores of CRPC-WNT. **c** Violin plot showing the module scores of CRPC-NE. **d** Violin plot showing the expression levels of *KLK3* in PCA1, PCA2 and PCA3 cell clusters. **e** Violin plot showing the expression levels of *NKX3-1* in PCA1, PCA2 and PCA3 cell clusters. **f** Feature plot showing the expression level of *ENO2*. **g** Cell composition across samples. **h** Progression trajectory on UMAP of tumor cells predicted by the slingshot package. **i** Pseudotime of tumor cells predicted by the *slingPseudotime* function implanted in slingshot package. **j** Violin plot showing the pseudotime of each tumor cell type.
