# Supplementary material for: LKB1 inactivation promotes epigenetic remodeling-induced lineage plasticity and antiandrogen resistance in prostate cancer
Source: Cell Res. 2025 Jan 2;35(1):59–71. doi: 10.1038/s41422-024-01025-z (PMC11701123; doi:10.1038/s41422-024-01025-z)
Supplement: Supplementary file 2 — Supplementary information, Fig. S2 [file 41422_2024_1025_MOESM2_ESM.pdf]

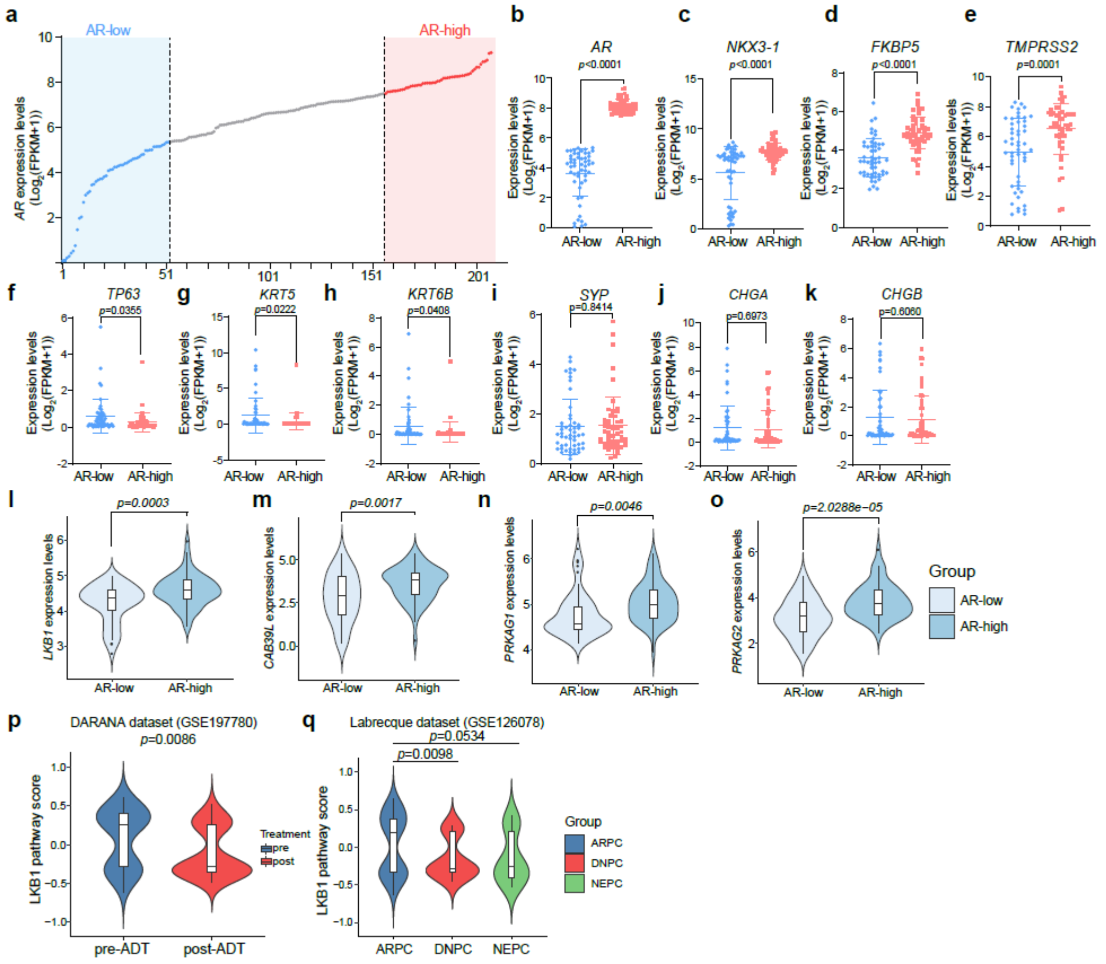

Supplementary information, Fig. S2. LKB1 pathway activity positively associates with AR expression. a Human prostate cancer samples are ranked and grouped according to *AR* expression levels. **b-e** The RNA expression levels of *AR* (**b**) and its target genes, *NKX3-1* (**c**), *FKBP5* (**d**) and *TMPRSS2* (**e**). **f-h** The RNA expression levels of basal markers, *TP63* (**f**), *KRT5* (**g**) and *KRT6B* (**h**). **i-k,** The RNA expression levels of neuroendocrine markers, *SYP* (**i**), *CHGA* (**j**) and *CHGB* (**k**). **l-o,** Violin plot showing the expression levels of the LKB1 pathway components, *LKB1* (**l**), *CAB39L* (**m**), *PRKAG1* (**n**) or *PRKAG2* (**o**) in AR-low and AR-high groups. **p** Box plot showing the LKB1 pathway scores in prostate tumors pre or post androgen-deprivation therapy in the DARANA dataset. **q** Box plot showing the LKB1 pathway scores in ARPC, DNPC and NEPC in the Labreque dataset.
