# Supplementary material for: LKB1 inactivation promotes epigenetic remodeling-induced lineage plasticity and antiandrogen resistance in prostate cancer
Source: Cell Res. 2025 Jan 2;35(1):59–71. doi: 10.1038/s41422-024-01025-z (PMC11701123; doi:10.1038/s41422-024-01025-z)
Supplement: Supplementary file 3 — Supplementary information, Fig. S3 [file 41422_2024_1025_MOESM3_ESM.pdf]

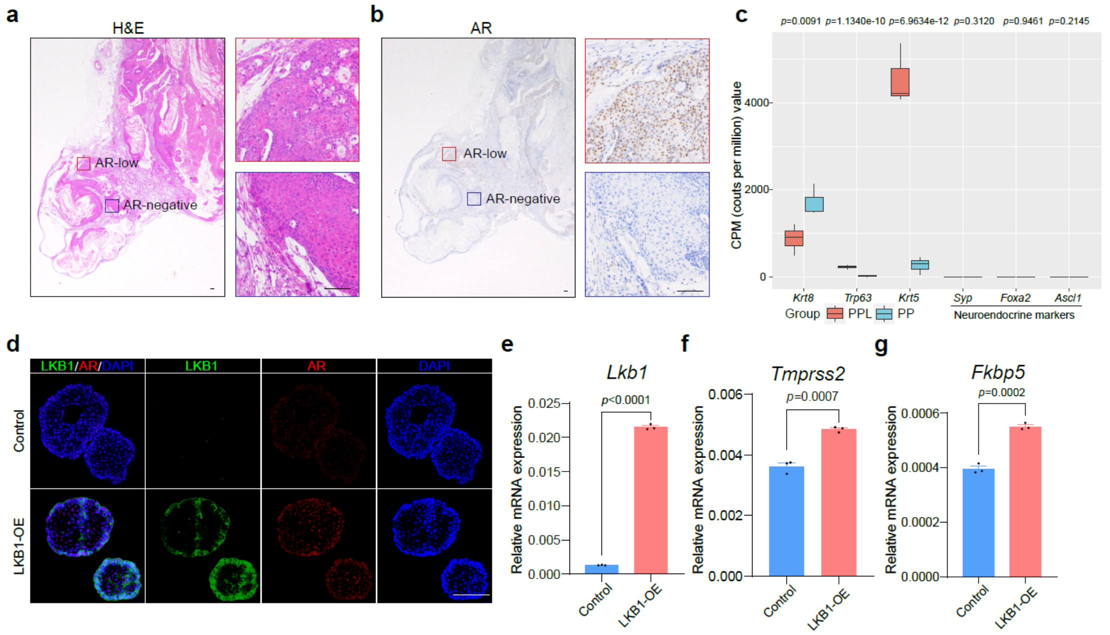

Supplementary information, Fig. S3. LKB1 loss promotes AR-negative lineage transition. **a** H&E staining of the prostate tumors from 15-week-old PPL mice. AR-low and AR-negative regions are labeled out. Scale bar represents 50 µm. **b** Immunohistochemical staining on AR of the prostate tumors from 15-week-old PPL mice. AR-low and AR-negative regions are labeled out. Scale bar represents 50 µm. **c** Box plot showing the RNA expression levels of *Krt8*, *Trp63*, *Krt5*, *Syp*, *Foxa2* and *Ascl1* in PPL and PP tumors. **d,** Immunofluorescence staining of LKB1, pAMPKα and DAPI in organoids derived from PPL primary tumor under Control or LKB1 overexpression (LKB1-OE) condition. Scale bar represents 50 µm. **e-f,** Box plot showing the relative mRNA expression levels of *Lkb1* (**e**), *Tmprss2* (**f**) and *Fkbp5* (**g**) quantified by qRT-PCR in the PPL tumor cells with (LKB1-OE) or without LKB1 overexpression (Control).
