# Supplementary material for: LKB1 inactivation promotes epigenetic remodeling-induced lineage plasticity and antiandrogen resistance in prostate cancer
Source: Cell Res. 2025 Jan 2;35(1):59–71. doi: 10.1038/s41422-024-01025-z (PMC11701123; doi:10.1038/s41422-024-01025-z)
Supplement: Supplementary file 4 — Supplementary information, Fig. S4 [file 41422_2024_1025_MOESM4_ESM.pdf]

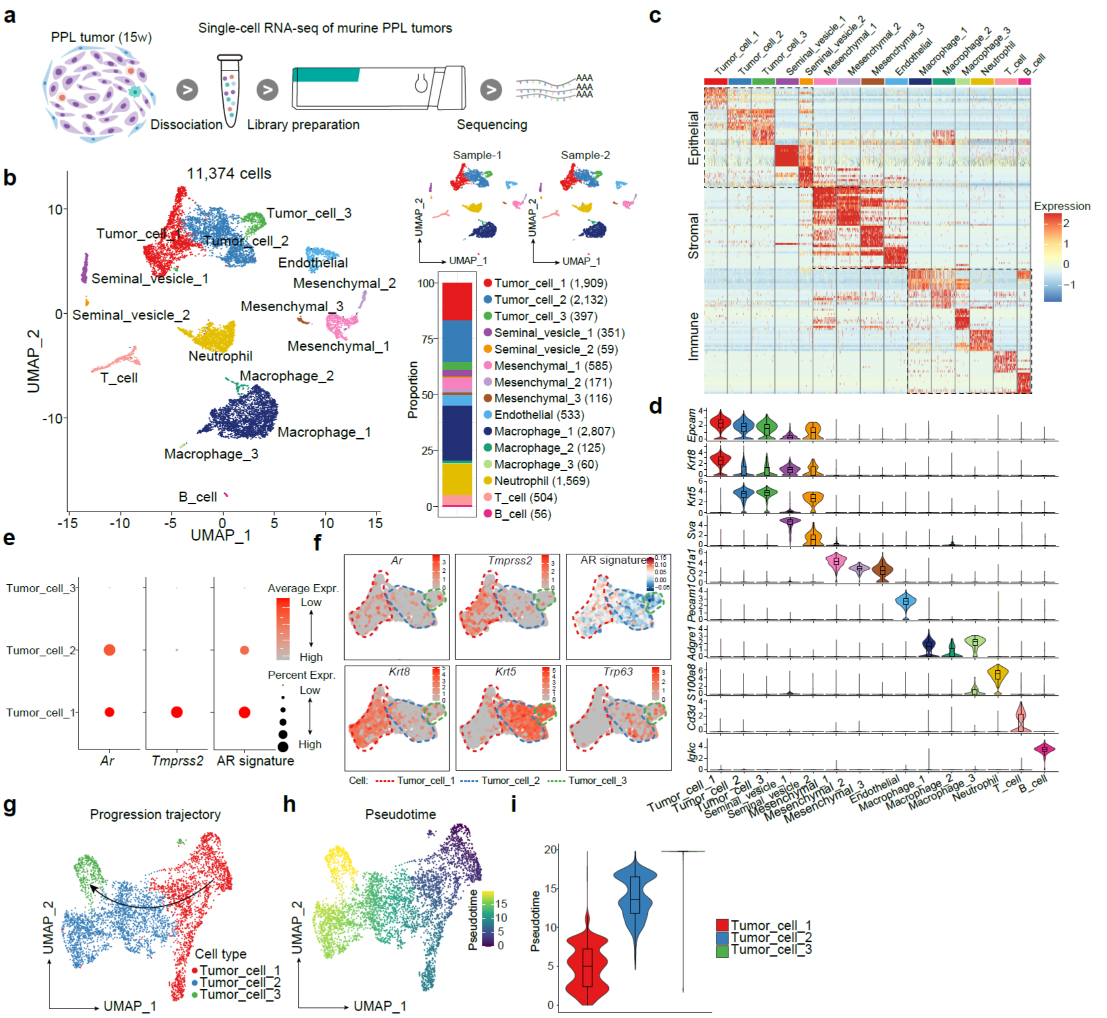

Supplementary information, Fig. S4. Single-cell RNA-seq of murine PPL tumors demonstrates the LKB1 loss-induced lineage plasticity. a Schematic of single-cell RNA-seq of murine PPL tumors. **b** UMAP showing the single-cell clusters across samples and bar plot showing cell proportion of each cluster. **c** Heatmap showing the RNA expression levels of the representative markers for each cell lineage. **d** Violin plot showing the RNA expression levels of the representative markers for each cell lineage. **e** Dot plot showing the RNA expression levels of *Ar* and *Tmprss2* as well as the AR signature scores across Tumor_cell_1, Tumor_cell_2 and Tumor_cell_2 clusters. **f** Feature plot showing the RNA expression levels of *Ar*, *Tmprss2*, *Krt8*, *Krt5* and *Trp63*, as well as the AR signature scores across Tumor_cell_1, Tumor_cell_2 and Tumor_cell_2 clusters. **g** Progression trajectory on UMAP of tumor cells predicted by slingshot package. **h** Pseudotime of tumor cells predicted by the *slingPseudotime* function implanted in slingshot package. **i** Violin plot showing the pseudotime of each tumor cell type.
