# Supplementary material for: LKB1 inactivation promotes epigenetic remodeling-induced lineage plasticity and antiandrogen resistance in prostate cancer
Source: Cell Res. 2025 Jan 2;35(1):59–71. doi: 10.1038/s41422-024-01025-z (PMC11701123; doi:10.1038/s41422-024-01025-z)
Supplement: Supplementary file 5 — Supplementary information, Fig. S5 [file 41422_2024_1025_MOESM5_ESM.pdf]

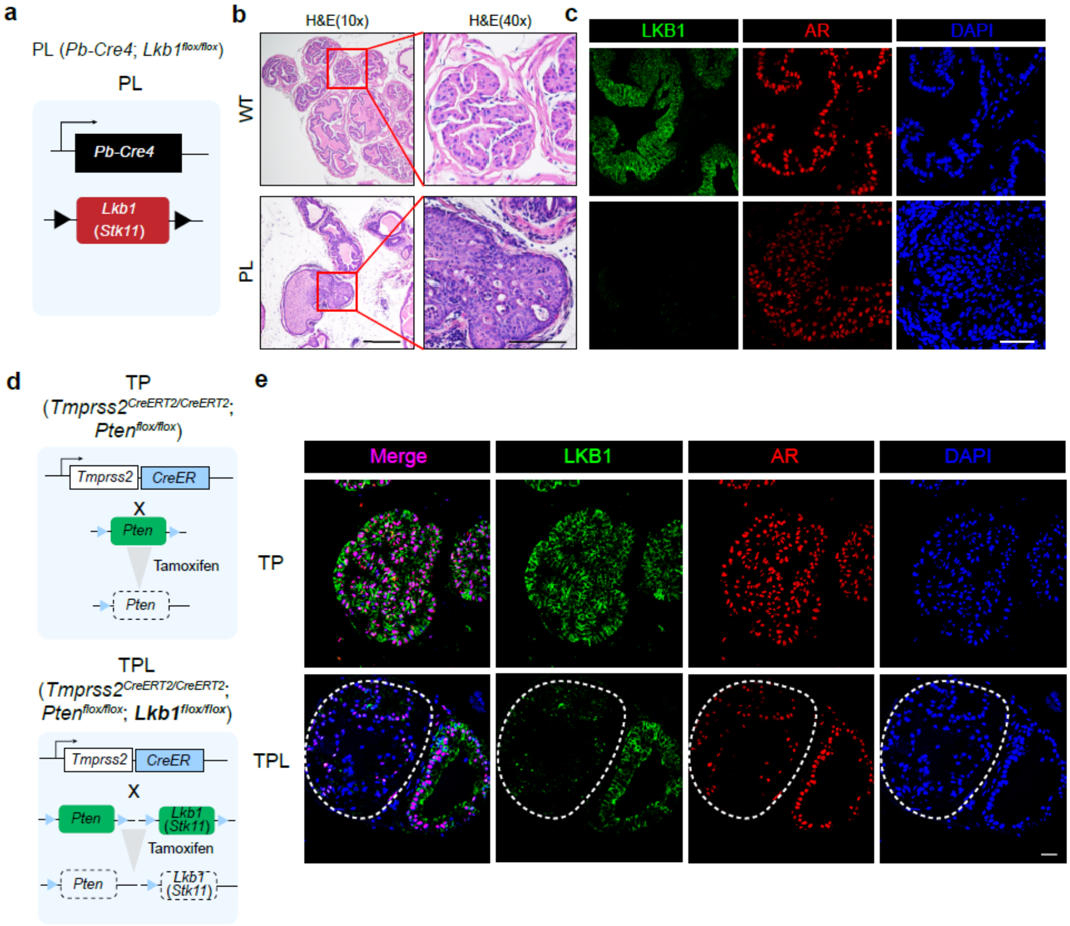

Supplementary information, Fig. S5. Two additional GEMM models confirms the suppressive effects of LKB1 loss on AR signaling. a Schematic of construction strategy of *Pb-Cre4*: *Lkb1*$^{\text{flox/flox}}$ (PL) mouse model. **b** H&E staining of the prostates of WT and PL mice. **c** Immunofluorescence staining of LKB1, AR and DAPI in the prostates of WT and PL mice. **d** Schematic of construction strategy of *Tmprss2*$^{\text{CreERT2/CreERT2}}$: *Pten*$^{\text{flox/flox}}$ (TP) and *Tmprss2*$^{\text{CreERT2/CreERT2}}$: *Pten*$^{\text{flox/flox}}$; *Lkb1*$^{\text{flox/flox}}$ (TPL) mouse models. **e** Immunofluorescence staining of LKB1, AR and DAPI in the prostates of TP and TPL mice. Scale bar represents 50 μm.
