# Supplementary material for: LKB1 inactivation promotes epigenetic remodeling-induced lineage plasticity and antiandrogen resistance in prostate cancer
Source: Cell Res. 2025 Jan 2;35(1):59–71. doi: 10.1038/s41422-024-01025-z (PMC11701123; doi:10.1038/s41422-024-01025-z)
Supplement: Supplementary file 6 — Supplementary information, Fig. S6 [file 41422_2024_1025_MOESM6_ESM.pdf]

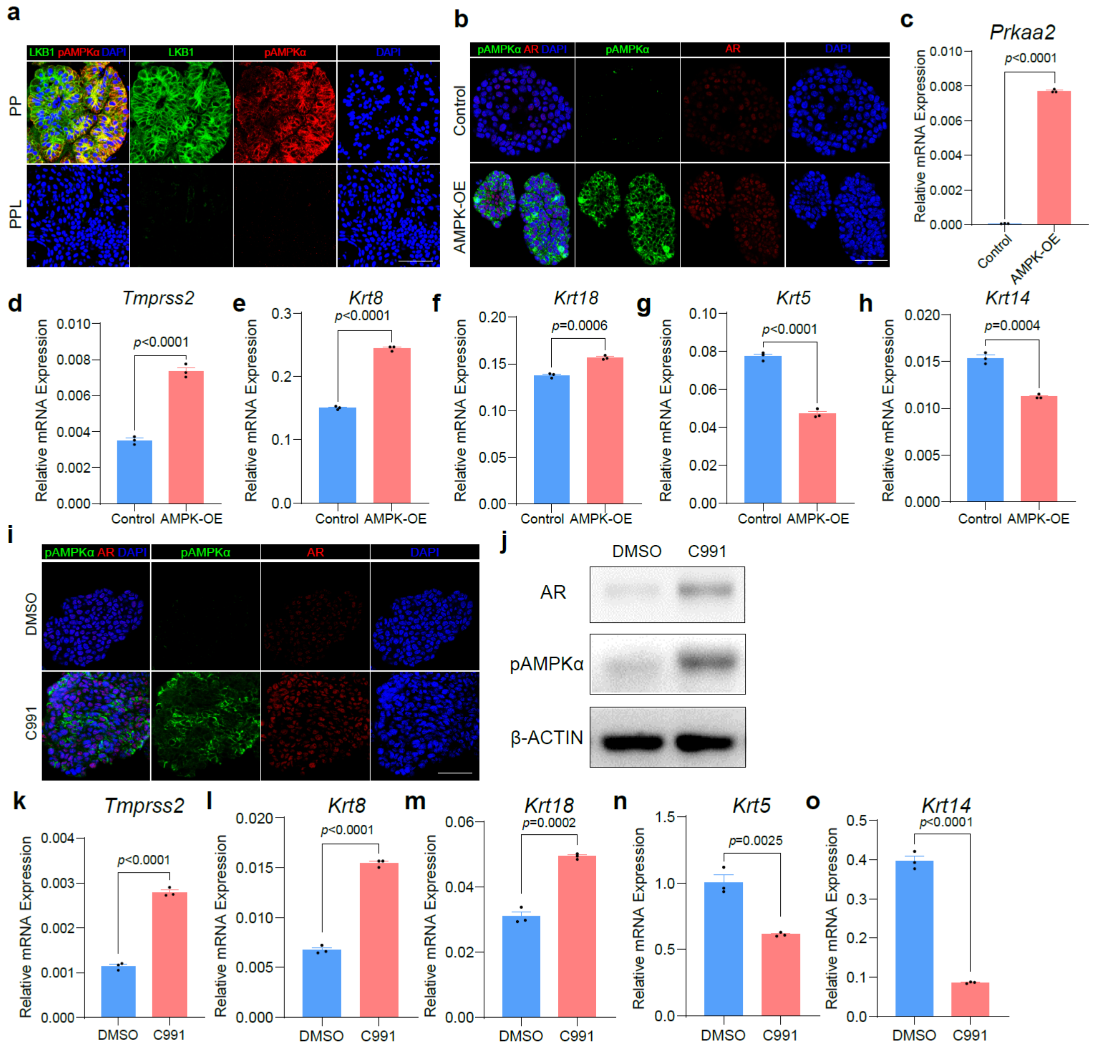

Supplementary information, Fig. S6. The effects of LKB1 loss on AR-low lineage plasticity are associated with AMPK. a Immunofluorescence staining of LKB1, pAMPKα and DAPI in the prostate tumor of 15-week-old PPL mice and 15-week-old PP mice. **b** Immunofluorescence staining of pAMPKα, AR and DAPI in PPL cancer cells with (AMPK-OE) or without (Control) AMPK overexpression. **c** Box plot showing the relative mRNA expression levels of *Prkaa2* quantified by qRT-PCR in PPL cancer cells with (AMPK-OE) or without (Control) AMPK overexpression. **d-h** Box plot showing the relative mRNA expression levels of *Tmprss2* (**d**), *Krt8* (**e**), *Krt18* (**f**), *Krt5* (**g**) and *Krt14* (**h**) quantified by qRT-PCR in PPL cancer cells with (AMPK-OE) or without (Control) AMPK overexpression. **j** Western blotting showing AR, pAMPKα and β-ACTIN expression levels in PPL cancer cells under DMSO or C991 treatment condition. **i** Immunofluorescence staining of pAMPKα, AR and DAPI in PPL cancer cells under DMSO or C991 treatment condition. **k-o** Box plot showing the relative mRNA expression levels of *Tmprss2* (**k**), *Krt8* (**l**), *Krt18* (**m**), *Krt5* (**n**) and *Krt14* (**o**) quantified by qRT-PCR in PPL cancer cells under DMSO or C991 treatment condition. Scale bar represents 50 μm.
