# Supplementary material for: LKB1 inactivation promotes epigenetic remodeling-induced lineage plasticity and antiandrogen resistance in prostate cancer
Source: Cell Res. 2025 Jan 2;35(1):59–71. doi: 10.1038/s41422-024-01025-z (PMC11701123; doi:10.1038/s41422-024-01025-z)
Supplement: Supplementary file 7 — Supplementary information, Fig. S7 [file 41422_2024_1025_MOESM7_ESM.pdf]

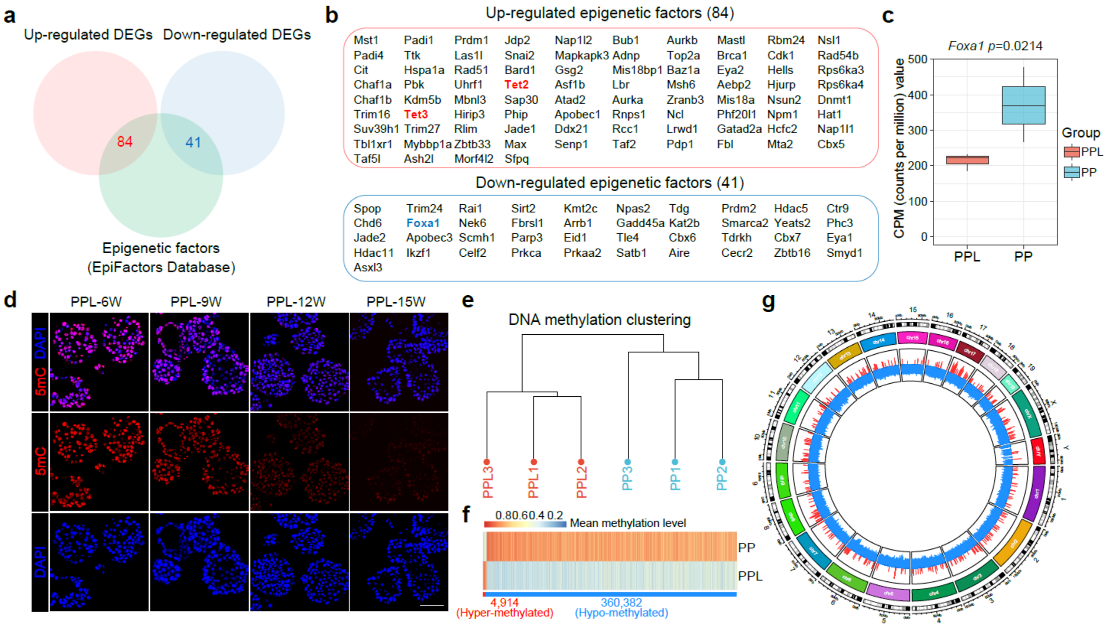

Supplementary information, Fig. S7. Identification of differentially expressed epigenetic factors reveals *Tet2* and *Tet3* as up-regulated epigenetic factors in PPL tumors. a Schematic showing the definition of differentially expressed epigenetic factors. **b** The differentially expressed epigenetic factors include 84 up-regulated epigenetic factors and 41 down-regulated epigenetic factors. **c** *Foxa1* is a down-regulated epigenetic factor, which is significantly down-regulated in PPL tumors. **d** Immunofluorescence staining of 5mC and DAPI in PPL-6W, -9W, -12W and -15W organoids. Scale bar represents 50 μm. **e** Clustering analysis of WGBS data of the prostate tumors of 15-week-old PP and PPL mice. **f** Heatmap showing the methylation levels of hypo-methylated and hyper-methylated DMLs in PPL and PP tumors. **g** Circos plot globally showing the hypo-methylated and hyper-methylated DMLs across all the chromosomes. Hypo-methylated DMLs are labelled by blue, and hyper-methylated DMRs are labelled by red respectively.
