# Supplementary material for: LKB1 inactivation promotes epigenetic remodeling-induced lineage plasticity and antiandrogen resistance in prostate cancer
Source: Cell Res. 2025 Jan 2;35(1):59–71. doi: 10.1038/s41422-024-01025-z (PMC11701123; doi:10.1038/s41422-024-01025-z)
Supplement: Supplementary file 8 — Supplementary information, Fig. S8 [file 41422_2024_1025_MOESM8_ESM.pdf]

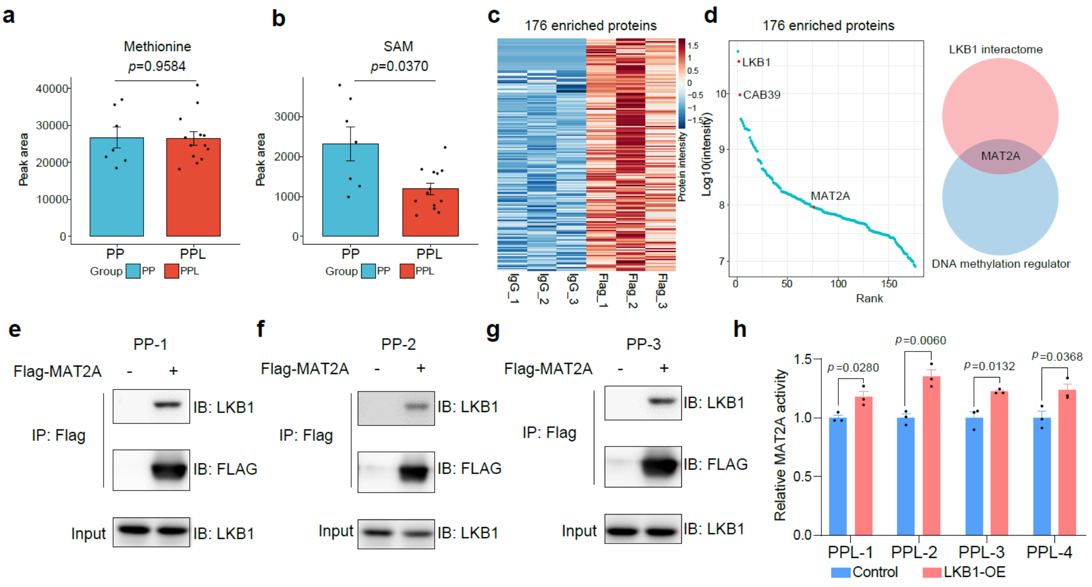

Supplementary information, Fig. S8. LKB1 loss attenuates the enzymatic activity of MAT2A, resulting in reduced SAM levels and consequent DNA hypomethylation. a, b Bar plot showing the levels of methionine (**a**) and SAM (**b**) in PPL and PP tumors quantified by peak area in HPLC-MS assay. **c** Heatmap showing the intensity of 176 proteins interacting with LKB1 identified by mass spectrometry. **d** Dot plot showing the 176 proteins interacting with LKB1 ranked by the protein intensity. **e-g** Co-IP demonstrating the physical interaction between LKB1 and MAT2A in PP-1 (**e**), PP -2 (**f**) and PP-3 (**g**) cancer cell lines. **h** Bar plot showing the relative MAT2A activity in multiple PPL cancer cell lines with (LKB1-OE) or without (Control) LKB1 overexpression.
