# Supplementary material for: LKB1 inactivation promotes epigenetic remodeling-induced lineage plasticity and antiandrogen resistance in prostate cancer
Source: Cell Res. 2025 Jan 2;35(1):59–71. doi: 10.1038/s41422-024-01025-z (PMC11701123; doi:10.1038/s41422-024-01025-z)
Supplement: Supplementary file 9 — Supplementary information, Fig. S9 [file 41422_2024_1025_MOESM9_ESM.pdf]

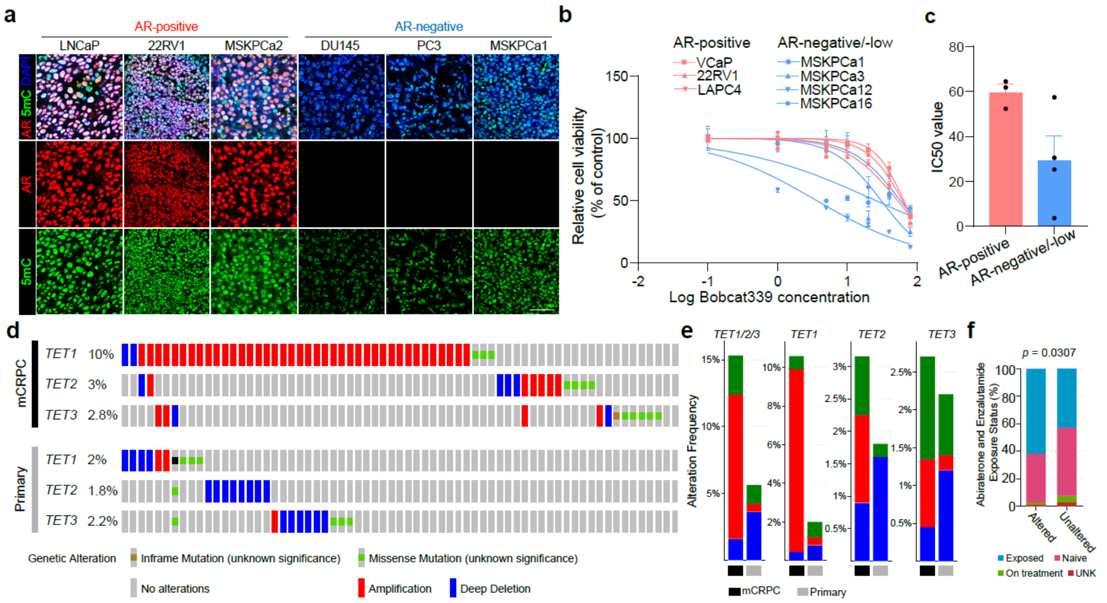

Supplementary information, Fig. S9. The genetic amplification of TETs is more frequent in mCRPC than in primary prostate cancer. a Immunofluorescence staining of 5mc, AR and DAPI in the xenografts of AR-positive cancer cells or organoids, LNCaP, 22RV1 and MSKPCa2, and AR-negative cancer cells or organoids, DU145, PC3 and MSKPCa1. Scale bar represents 50 µm. **b**, **c** Drug sensitivity assay of Bobcat339 in AR-positive prostate cancer cells and AR-low/negative prostate cancer cells (**b**) and its quantification of IC50 value (**c**). **d** OncoPrint plot showing the genetic alteration of *TET1*, *TET2* and *TET3* in mCRPC and primary prostate cancer. **e** Bar plot showing the alteration frequency of *TET1*, *TET2* and *TET3* in mCRPC and primary prostate cancer. **f** TETs alteration is associated with the exposure to abiraterone and enzalutamide.
