# Supplementary material for: LKB1 inactivation promotes epigenetic remodeling-induced lineage plasticity and antiandrogen resistance in prostate cancer
Source: Cell Res. 2025 Jan 2;35(1):59–71. doi: 10.1038/s41422-024-01025-z (PMC11701123; doi:10.1038/s41422-024-01025-z)
Supplement: Supplementary file 17 — Supplementary information, Data S1 [file 41422_2024_1025_MOESM17_ESM.pdf]

Supplementary information, Data S1

Supplementary Materials and methods

Histological and immunostaining assays

Mouse prostate tissues were fixed with 4% paraformaldehyde overnight at 4 °C as previously reported. Fixed tissues were washed with cold PBS for three times and then dehydrated sequentially with 75%, 95%, and 100% ethanol for 1.5 hours each. Tissues were cleared in xylene for 22 minutes at 55 °C and then immersed in paraffin 3 times for 2 hours each at 65 °C. Paraffin-embedded tissues were cut into 2-mm-thick sections and stored at room temperature. For immunohistochemistry, freshly sliced 2-micron paraffin sections were dewaxed and subjected to antigen retrieval by boiling for 45 minutes in 0.01 M citrate buffer. Endogenous peroxidase activity was quenched by immersing the slides in 3% H_2O_2 for 20 minutes. The slides were then blocked in 2% BSA for 1 hour and stained with primary antibodies in blocking buffer at 4 °C overnight or at room temperature for 2 hours. The slides were then incubated with an HRP-conjugated secondary antibody (OriGene) for 20 minutes at room temperature (RT) and stained with DAB (Vector Laboratories). The following primary antibodies were used: anti-Ar (Abcam, ab108341, 1:2000), anti-Lkb1 (CST, 13031S, 1:500), anti-Trp63 (Abcam, ab735, 1:500), anti-CK8 (Covance, MMS-162-P, 1:100). For immunofluorescence, sections were dewaxed and subjected to antigen retrieval by boiling for 45 minutes in 0.01 M citrate buffer. Then, sections were blocked in 5% goat serum for 1 hour at RT, stained with primary antibodies at 4 °C overnight, washed with PBST three times and incubated with secondary antibodies for 1 hour at RT. Sections were then washed with PBST for three times and stained with DAPI (Thermo) for 7 minutes. The following primary antibodies were used: anti-AR (Abcam, ab108341, 1:2000), anti-LKB1 (CST, 13031S, 1:500), anti-P63 (Abcam, ab735, 1:500), anti-CK8 (Covance, MMS-162-P, 1:100), anti-5mC (Abcam, ab10805, 1:500), anti-Cleaved caspase-3 (CST, 9664S, 1:200), anti-TMPRSS2 (Abcam, ab92323).

Cell culture

HEK 293T cells (CRL-1573) were from ATCC and cultured in the CO_2 incubator (5% CO_2, 37 °C) with DMEM supplemented with 10% (v/v) fetal bovine serum (EXCELL)

and penicillin (100 U/mL)/streptomycin (0.1 mg/mL). PP and PPL tumor cell was cultured in the CO_2 incubator (5% CO_2, 37 °C) with mouse prostate culture medium which was consisted of Advanced DMEM/F12 medium supplemented with 10 mM HEPES (Gibco), 2 mM GlutaMAX-1 (Gibco), 500× Primocin (InvivoGen), P/S, 1× B27 (Gibco), 1.56 mM N-acetylcysteine (Sigma), 0.5 mM A83-01 (TOCRIS), 10 mM Y27632 (Selleck), EGF (Invitrogen), 1 nM DHT (Sigma), 10% in-house-prepared R-Spondin1 and 10% Noggin. For LNCaP and 22Rv1, which was cultured in the CO_2 incubator (5% CO_2, 37 °C) with RPMI Medium 1640 basic (1×) supplemented with 10% (v/v) fetal bovine serum (EXCELL) and penicillin (100 U/mL)/streptomycin (0.1 mg/mL). DU145 was cultured in the CO_2 incubator (5% CO_2, 37 °C) with DMEM supplemented with 10% (v/v) fetal bovine serum (EXCELL) and penicillin (100 U/mL)/streptomycin (0.1 mg/mL).

Lentivirus production and transduction of cell

Lentivirus was produced by 293T cell which was transfected with target plasmid and packaging plasmids by the manufacturer's protocol of Vigofect transfection reagent (Vigorous Biotectnology, T001). The supernatant was collected at 48 hours and 60 hours post transfection, filtered through a 0.45 μm cell strainer and concentrated by an ultracentrifugation at 27000 rpm for 2 hours. LNCaP and 22Rv1 cells were digested into single cell and seeded into 6-well cell culture plate 12 hours prior to transduction. Concentrated lentivirus was transduced into cells with polybrene by a centrifugation at 500× *g* for 55 min at room temperature. For the AMPK overexpression assay, the plasmid of murine *Prkaa2* was purchased from MIAOLING BIOLOGY (P43724). For gene knockdown plasmids. The shRNA hairpins for targeting *LKB1* were listed below:

sh*LKB1*-1:
TGCTGTTGACAGTGAGCGCCCAGCTGGTGGATGTGTTATATAGTGAAGCCACAGATGTATATAACACATCCACCAGCTGGATGCCTACTGCCTCGGA;

sh*LKB1*-2:
TGCTGTTGACAGTGAGCGACCGTCAAGATCCTCAAGAAGATAGTGAAGCCACAGATGTATCTTCTTGAGGATCTTGACGGCTGCCTACTGCCTCGGA;

sh*LKB1*-3:

TGCTGTTGACAGTGAGCGAGGCCGTCAAGATCCTCAAGAATAGTGAAGCC ACAGATGTATTCTTGAGGATCTTGACGGCCCTGCCTACTGCCTCGGA.

qRT-PCR

Total RNA was extracted from cells or tissues with TRIzol (Ambion, 15596018). The solution was mixed by pipetting and left to lyse for 30 minutes at room temperature. Next, 1/5 volume of chloroform was added and vortexed for 15 seconds. After a 2-minute incubation at room temperature, the mixture was centrifuged at 13,000× *g* for 15 minutes at 4 °C. The aqueous phase was transferred into a new tube, and an equal volume of isopropanol was added. Following centrifugation at 13,000× *g* for 10 minutes at 4 °C, the supernatant was discarded, and the pellet was resuspended in 75% ethanol. Another centrifugation at 13,000× *g* for 7 minutes at 4 °C was performed. The supernatant was carefully removed and discarded, and the pellet was resuspended in 40 μL of nuclease-free water. Reverse transcription was further carried out using PrimeScriptTM RT Master Mix (TaKaRa, RR036A) with 400 ng of total RNA as input. qRT-PCR was conducted using SYBR qPCR Mix (YEASEN, 1184ES08) according to the manufacturer's protocol. The primer sequences are as follows:

Actb-F: 5'-CATTGCTGACAGGATGCAGAAGG-3';
Actb-R: 5'-TGCTGGAAGGTGGACAGTGAGG-3';
Lkb1-F: 5'-GCCTGGAATACCTACACAGCCA-3';
Lkb1-R: 5'-GCAGGTGTCATCCACAGCGAAA-3';
Prkaa2-F: 5'-CTGAAGCCAGAGAATGTGCTGC-3';
Prkaa2-R: 5'-GAGATGACCTCAGGTGCTGCAT-3';
Tmprss2-F: 5'-AAGTCCTCAGGAGCACTGTGCA-3';
Tmprss2-R: 5'-CAGAACCTCCAAAGCAAGACAGC-3';
Fkbp5-F: 5'-GATTGCCGAGATGTGGTGTTCG-3';
Fkbp5-R: 5'-GGCTTCTCCAAAACCATAGCGTG-3';
Krt8-F: 5'-TGGAAGGACTGACCGACGAGAT-3';
Krt8-R: 5'-GGCACGAACTTCAGCGATGATG-3';
Krt18-F: 5'-AATCAGGGACGCTGAGACCACA-3';
Krt18-R: 5'-GCTCCATCTGTGCCTTGTATCG-3';
Krt5-F: 5'-GAACAGAGGCTGAGTCCTGGTA-3';

Krt5-R: 5'-TCTCAGCCTCTGGATCATTCGG-3';
Krt14-F: 5'-GAAGAACCGCAAGGATGCTGAG-3';
Krt14-R: 5'-TGCAGCTCGATCTCCAGGTTCT-3'.

Western blotting

Cell lysates were prepared using RIPA buffer supplemented with proteinase and phosphatase inhibitors. Protein concentration was measured using a BCA assay (Thermo). Fifteen micrograms of protein were separated by SDS-PAGE and transferred onto a 0.45mm PVDF membrane (GE). The membrane was blocked for 1 hour at room temperature in TBST buffer with 5% milk, then incubated with primary antibodies diluted in TBST buffer with 5% milk either overnight at 4 °C or for 2 hours at room temperature. Subsequently, the membrane was incubated with rabbit HRP-conjugated secondary antibodies (SAB, #L3012) in TBST buffer with 5% milk for 1 hour at room temperature. The primary antibodies used included anti-β-ACTIN (Sigma-Aldrich, A3854, 1:5000), anti-AR (Abcam, ab108341, 1:2000), anti-p-AMPKa (T172) (CST, 2535S, 1:1000), anti-LKB1 (CST, 13031S, 1:1000), anti-Flag (Proteintech 20543-1-AP) antibodies.

Prostate cell dissociation

Mouse prostate was dissociated and transferred into 1.5 mL tube containing enzyme mix (0.75 mL of DMEM/F12 buffer with 1× collagenase/hyaluronidase) after removing connective tissue. Prostate was cut into small pieces and put on a thermos-shaker for an incubation step up to 1500 rpm for 30 minutes at 37 ℃. The dissociated tissue was centrifuged at 1700 rpm for 3 minutes and then the supernatant was removed. Prostate tissue was further dissociated with TrypLE (Gibco, no. 12605-028) for 15 minutes at 37 ℃ on the thermos-shaker. The digestive reaction was quenched by adding 1 mL of DMEM supplemented with 10% FBS and the cell suspension was applied to a 70 μm cell strainer placed on a new 15 mL tube. Cell strainer was then washed with 2 mL of DMEM supplemented with 10% FBS. Centrifugation at 1700 rpm for 3 minutes was performed to collect cells.

Organoid culture

PP and PPL mouse prostate tumor was freshly dissociated into single cells. Count the cells and in total 500 cells were seeded in a 50 µL of Matrigel drop in the middle of one well of 24-well suspension plate. The culture plate was placed into the CO_2 incubator (5% CO_2, 37 °C) for 15 minutes, allowing the Matrigel to solidify. 1 mL mouse prostate culture medium was gently pipetted into each well and then the plate was placed into CO_2 incubator (5% CO_2, 37 °C).

Mass spectrometry analysis of LKB1-associated proteins

Flag-LKB1 proteins were immunoprecipitated from PP cells using anti-Flag agarose beads. The beads were washed five times with PBS and then processed as follows:

On-beads Digestion

Beads were dissolved 50 µL of 8 M urea with 100 mM Tris-Cl (pH 8.5). 5 mM Tris(2-Carboxyethyl)-Phosphine HCl (TCEP, Thermo Scientific) for reduction and 10 mM iodoacetamide (IAA, Sigma) for alkylation were added, sonicated and incubated at room temperature in dark for 30 minutes, respectively. The protein mixture was diluted four times with 100 mM Tris-Cl and digested with Trypsin at 1:50 (w/w) (Promega). The digestion was stopped by 5% Formic Acid (FA, Thermo Scientific), and the peptide mixture was desalted by MonoSpinTM C18 column (GL Science). Desalted mixture was dried with a SpeedVac and resuspended in 0.1% FA for MS analysis.

HPLC-tandem MS (MS/MS) analysis of peptides

The peptide mixture was analyzed by a home-made 30 cm-long pulled-tip analytical column (75 µm ID packed with ReproSil-Pur C18-AQ 1.9 µm resin, Dr. Maisch GmbH), the column was then placed in-line with an Easy-nLC 1200 nano HPLC (Thermo Scientific) for mass spectrometry analysis. The analytical column temperature was set at 55 °C during the experiments. The mobile phase and elution gradient used for peptide separation were as follows: 0.1% formic acid in water as buffer A and 0.1% formic acid in 80% acetonitrile as buffer B, 0-1 min, 3%-8% B; 1-191 min, 8-25% B; 191-219 min, 25%-50% B, 219-220 min, 50%-100% B, 220-240 min, 100% B. The flow rate was set

as 300 nL/min.

Mass Spectrometry

Data-dependent MS/MS analysis was performed with a Q Exactive Orbitrap mass spectrometer (Thermo Scientific). Peptides eluted from the LC column were directly electrosprayed into the mass spectrometer with the application of a distal 2.5-kV spray voltage. A cycle of one full-scan MS spectrum (m/z 300-1800) was acquired followed by top 20 MS/MS events, sequentially generated on the first to the twentieth most intense ions selected from the full MS spectrum at a 30% normalized collision energy. Full scan resolution was set to 70,000 with automated gain control (AGC) target of 3e6. MS/MS scan resolution was set to 17,500 with isolation window of 1.8 m/z and AGC target of 1e5. The number of microscans was one for both MS and MS/MS scans and the maximum ion injection time was 50 and 100 ms, respectively. The dynamic exclusion settings used were as follows: charge exclusion, 1 and >8; exclude isotopes, on; and exclusion duration, 30 seconds. MS scan functions and LC solvent gradients were controlled by the Xcalibur data system (Thermo Scientific).

Data Analysis

The acquired MS/MS data were analyzed against a mouse database using the software MaxQuant. Trypsin was defined as cleavage enzyme; Cysteine alkylation by iodoacetamide was specified as fixed modification with mass shift 57.02146; Methionine oxidation was set as dynamic modification with mass shift 15.9949.
